# A diterpene synthase from the sandfly *Lutzomyia longipalpis* produces the pheromone sobralene

**DOI:** 10.1073/pnas.2322453121

**Published:** 2024-03-12

**Authors:** Charles Ducker, Cameron Baines, Jennifer Guy, Antônio Euzébio Goulart Santana, John A. Pickett, Neil J. Oldham

**Affiliations:** ^a^School of Chemistry, University of Nottingham, University Park, Nottingham NG7 2RD, United Kingdom; ^b^Center of Engineering and Agrarian Science, Federal University of Alagoas, Maceio 57100-000, Brazil; ^c^School of Chemistry, Cardiff University, Cardiff CF10 3AT, United Kingdom

**Keywords:** terpene biosynthesis, diterpene synthase, sex/aggregation pheromone, sobralene

## Abstract

The phlebotomine sandfly, *Lutzomyia longipalpis*, a major vector of the *Leishmania* parasite, uses terpene pheromones to attract conspecifics for mating. Examination of the *L. longipalpis* genome revealed a putative terpene synthase (TPS), which—upon heterologous expression in, and purification from, *Escherichia coli*—yielded a functional enzyme. The TPS, termed *Ll*TPS, converted geranyl diphosphate (GPP) into a mixture of monoterpenes with low efficiency, of which β-ocimene was the major product. (*E,E*)-farnesyl diphosphate (FPP) principally produced small amounts of (*E*)-β-farnesene, while (*Z,E*)- and (*Z,Z*)-FPP yielded a mixture of bisabolene isomers. None of these mono- and sesquiterpenes are known volatiles of *L. longipalpis*. Notably, however, when provided with (*E,E,E*)-geranylgeranyl diphosphate (GGPP), *Ll*TPS gave sobralene as its major product. This diterpene pheromone is released by certain chemotypes of *L. longipalpis*, in particular those found in the Ceará state of Brazil. Minor diterpene components were also seen as products of the enzyme that matched those seen in a sandfly pheromone extract.

## Significance

Terpenes are widely used in nature for chemical communication, but our understanding of how these structurally diverse natural products are produced by insects is only now beginning to emerge. Males of the sandfly, *Lutzomyia longipalpis*, use terpene pheromones to lure females and other males to mating sites. This insect attracts considerable attention due to its role as a vector for the *Leishmania* parasite, which causes the neglected tropical disease leishmaniasis. In this study, a diterpene synthase that produces the pheromone component sobralene is identified, heterologously expressed and functionally characterized. This represents identification of a terpene synthase (TPS) from *Lutzomyia* and shows that insects are capable of biosynthesizing diterpenes. It offers the potential for sustainable production of this compound through biocatalysis.

**Fig. 1.**
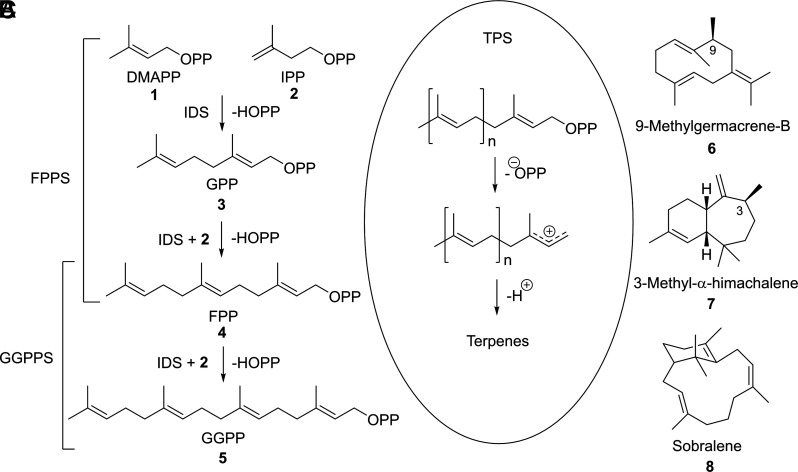
The origin of terpene natural products. (*A*) Isoprenyl diphosphate synthesis from DMAPP (**1**) and IPP (**2**) catalyzed by IDS enzymes, including FPPS and GGPPS, and leading to FPP (**4**) and GGPP (**5**). (*B*) Terpene synthesis via carbocation intermediates catalyzed by the action of TPS enzymes. (*C*) Structures of the terpene pheromones of *L. longipalpis*, (*S*)-9-methylgermacrene-B (**6**), (1*S*,3*S*,7*R*)-3-methyl-α-himachalene (**7**) and sobralene (**8**).

Terpenes, a class of isoprenoids, comprise a highly diverse group of natural products often possessing pronounced biological activity (1). Within the eukaryotes, plants and fungi are recognized as the major producers, but amoebae and insects also utilize terpenes for a range of ecological functions (2, 3). Terpenes are biosynthesized by two main routes: the mevalonate (MVA) pathway and the methylerythritol phosphate (MEP) pathway (4, 5), with both resulting in the production of dimethylallyl diphosphate (DMAPP, **1**) and isopentenyl diphosphate (IPP, **2**), which are the C_5_ building blocks of terpene hydrocarbons and the wider class of isoprenoid metabolites (Fig. 1). It is believed that animals are only capable of employing the MVA pathway, while plants, for example, can utilize both the MVA and MEP routes (6). DMAPP and IPP are assembled into chains of varying lengths by a class of enzymes known as isoprenyl diphosphate synthases (IDSs). DMAPP plus one unit of IPP produces geranyl diphosphate (GPP, C_10_, **3**), DMAPP plus two units of IPP yields farnesyl diphosphate (FPP, C_15_, **4**), and DMAPP plus three units of IPP gives geranylgeranyl diphosphate (GGPP, C_20_, **5**) (Fig. 1). IDSs responsible for producing a specific length of isoprenyl chain are often named for their product, e.g., farnesyl diphosphate synthase (FPPS), geranylgeranyl diphosphate synthase (GGPPS), etc. Terpenes may then be produced by the action of terpene synthases (TPSs) upon isoprenyl diphosphates via removal of inorganic diphosphate (PPi) leading to an allyl cation intermediate (Fig. 1) (7, 8). Controlled rearrangement and/or elimination of a proton from this species can result in one or several products from a potentially vast array of cyclic and acyclic structures, depending upon the environment within a particular TPS active site.

Although many TPSs derived from plants and microorganisms have been identified, far fewer examples from insects are known. In a pioneering study, Beran et al. in this journal (2016) showed that insect TPSs appear to have evolved from IDS ancestors through gene duplication (9). Thus, in insects, TPSs are much closer to IDSs than is the case in plants, fungi, or bacteria. Recently, Rebholtz et al. analyzed sequences of putative or confirmed IDS/TPS genes from eight insect orders to produce a comprehensive phylogeny (10). From this work, a tendency for a greater number of amino acid substitutions in IPP-binding motifs (IBMs) has been shown to be associated with the evolution from IDSs to TPSs, which appears to have occurred independently across the insects. Examination of the number of amino acid substitutions in IBMs is emerging as a useful tool in identifying candidate TPSs from insect genomic data.

The phlebotomine sandfly *Lutzomyia longipalpis* is a vector for the *Leishmania* parasite, the causative pathogen of the neglected tropical disease leishmaniasis (11). Although it should be noted that it is more correct to use the plural, leishmaniases, as this is a group of diseases with a range of symptoms. Cutaneous leishmaniasis is the most common form globally, and results in disfiguring skin ulcers, whereas visceral leishmaniasis, which targets the body’s internal organs, is the most severe form, being invariably fatal if untreated (12, 13). Male sandflies produce sex/aggregation pheromones, which they use to attract both females and males to lek mating sites (14). There are several defined geographical chemotypes of *L. longipalpis* in Brazil (15), which are associated with differing pheromone chemistry: those that produce the homosesquiterpenes (*S*)-9-methylgermacrene-B (**6**) or (1*S*,3*S*,7*R*)-3-methyl-α-himachalene (**7**), those that employ diterpenes, of which sobralene (**8**) is the only structurally characterized member, and those that utilize a mixture of these homosesquiterpene (characterized originally by Pickett) and diterpene components (16–21). Control of the sandfly is a recognized means of leishmaniasis prevention, and the hope is that pheromones may be used to assist mass trapping. Hamilton and colleagues have shown that (±)-9-methylgermacrene-B, produced by chemical means (22), is, indeed, effective for trapping *L. longipalpis* in the field (23–26). Due to their structural complexity, there remain, however, significant barriers to the effective synthetic production of the three pheromone components for use in widespread insect control. This consideration, together with the interesting chemical structures of these terpenes, has motivated us to explore their biosynthesis in the insect and to identify TPS enzymes responsible for pheromone production.

Here, we report the identification, expression, and functional characterization of a terpene synthase from *L. longipalpis* that is capable of producing the pheromone component sobralene (**8**) from (*E,E,E*)-GGPP. This shows that insects are capable of true diterpene biosynthesis. It is hoped that these findings may offer a potential route to sustainable production of the isomerically pure compound.

## Results

### Identification of a Putative Terpene Synthase from the *L. longipalpis* Genome.

**Fig. 2.**
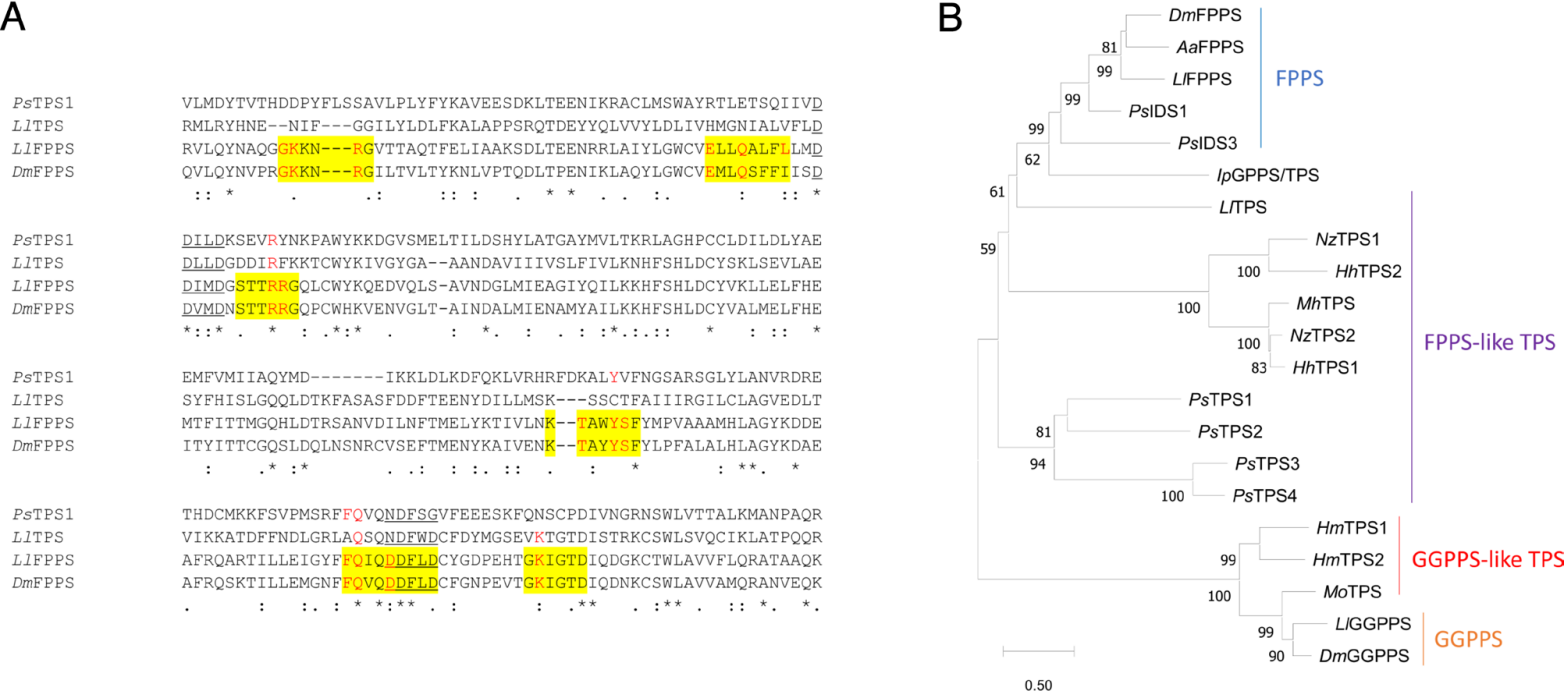
Sequence analysis of insect IDS and TPS enzymes. (*A*) Muscle alignment of XP_055677521.1 (*Ll*FPPS, aa108-341) and XP_055691875.1 (*Ll*TPS, aa34-263), compared against the FPPS of *Drosophila melanogaster* (*Dm*FPPS, aa116-349) and TPS1 from *Phyllotreta striolata* (*Ps*TPS1, aa75-307). Underlined residues denote location of DDxxD motifs, with yellow highlighting predicted IPP-binding motifs (IBMs) in IDS enzymes and red identifying conserved residues reported to make direct contacts with IPP. (*B*) Phylogenetic tree of selected insect IDS and TPS enzymes inferred by maximum-likelihood analysis.

Examination of the published *L. longipalpis* genome revealed the presence of several candidate IDS/TPS genes (27). Of these, XP_055691875.1, which is annotated in the genome as a farnesyl diphosphate synthase-like gene, attracted our particular interest as, when compared to FPPSs, it showed significant amino acid substitutions in the IPP-binding motifs (IBMs) characterized by Rebholtz et al. The *L. longipalpis* FPPS, *Ll*FPPS, displayed full conservation of all 15 IPP-interacting residues (IBMs 1 to 5 and SARM, Fig. 2). This protein is annotated as an FPPS in the published *L. longipalpis* genome and functionally confirmed as such in this study (*SI Appendix*, Figs. S1 and S2). In contrast, XP_055691875.1 retained only 3 of the 15, which made it a strong TPS candidate. This result was comparable to a characterized sesquiterpene TPS from the flea beetle *Phyllotreta striolata*, *Ps*TPS1, which maintained 4 of the 15 conserved residues (Beran et al.). Additionally, the sequence of XP_055691875.1 showed high identity with an RNA contig, identified as C94, from a transcriptomic study of the *L. longipalpis* pheromone gland (28).

### Expression and Functional Testing of Putative TPS.

The gene for XP_055691875.1 was synthesized commercially in pET100/D-TOPO plasmid, and soluble protein successfully expressed and purified from *Escherichia coli* as described in *Materials and Methods* (*SI Appendix*, Fig. S2). Native electrospray–mass spectrometry of the enzyme showed that it was homodimeric in structure (predicted monomer mass 43,140 Da and measured mass 86,365 Da, *SI Appendix*, Fig. S2*C*). Although this is not a surprising result given the similarity between insect TPSs and FPPSs and the fact that FPPSs are known to be dimeric, it does confirm the quaternary structure of this class of insect TPSs. The difference between the predicted polypeptide dimer mass (86,280 Da) and the measured value was 85 Da, which was likely due to copurification of 3 to 4 Mg^2+^ ions with the protein. The activity of the putative TPS was assayed by incubation with a panel of isoprenyl diphosphates (Table 1) followed by GC–MS analysis of the resulting volatiles. It was clear from these results that the protein product of gene XP_055691875.1 was, indeed, a TPS and that it was capable of producing mono-, sesqui-, and diterpenes from the appropriate precursors. For this reason, from this point in the paper, the enzyme is referred to as *Ll*TPS. IDS and TPS enzymes typically use one or more Mg^2+^ ions as a cofactor, which both bind and activate the diphosphate moiety. Occasionally, other divalent cations are employed (29, 30), but—of the divalent metal ion cofactors tested—Mg^2+^ gave the greatest product yield. Co^2+^ and Mn^2+^ resulted in an approximate factor-10 and factor-100 decrease in terpene products, respectively. If no metal ions were added, no activity was seen. Each metal ion produced a broadly similar profile of products. GPP was converted to small quantities of myrcene, limonene, and (*E*)- and (*Z*)-β-ocimene as the only true terpenes, with linalool and geraniol also present (Table 1 and *SI Appendix*, Table S1 and Figs. S3 and S4). (*E,E*)-FPP also yielded weak signals, the largest of which was identified as (*E*)-β-farnesene (Table 1 and *SI Appendix*, Table S1 and Figs. S5*A* and S6). Minor peaks due to α-, β-, and γ-bisabolenes were also seen. In contrast, (*Z,E*)- and (*Z,Z*)-FPP, the metabolically less familiar and generally less abundant isomers of FPP, gave significant quantities of bisabolenes. The major product from (*Z,E*)-FPP was (*Z*)-γ-bisabolene (ca. 50% of the total), with β-bisabolene, (*E*)-α-bisabolene, and (*Z*)-α-bisabolene constituting the remaining 50 % (Table 1 and *SI Appendix*, Table S1 and Figs. S5*B* and S6). (*Z,Z*)-FPP yielded β-bisabolene as the major product (ca. 70 %) with minor amounts of (*E*)-α-bisabolene and (*E*)-γ-bisabolene (Table 1 and *SI Appendix*, Table S1 and Figs. S5*C* and S6). Additionally, a GC peak with an EI mass spectrum very similar to that of helminthogermacrene was seen. No germacrene-B, γ-elemene, or himachalenes (*SI Appendix*, Figs. S9 and S10) were seen as products from any of the FPP isomers.

**Table 1.** Terpene synthase (TPS) activity of *Ll*TPS from *L. longipalpis* when incubated with a panel of isoprenyl diphosphate substrates in the presence of Mg^2+^

Substrate	Terpene product	
Geranyl diphosphate	β-Myrcene	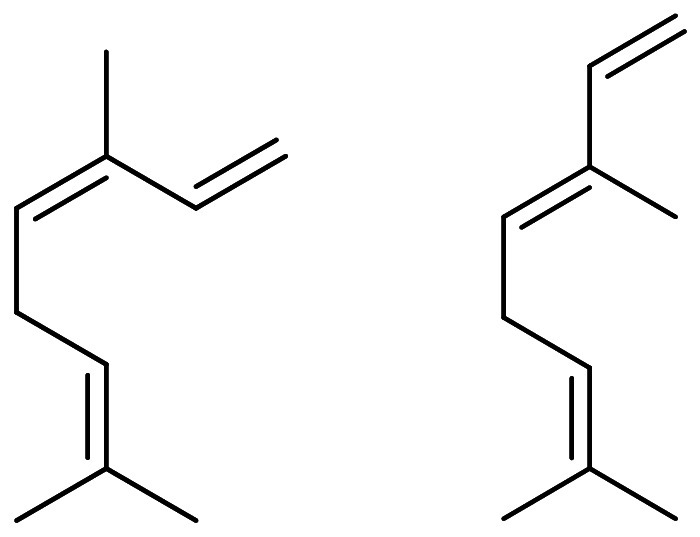
	Limonene	
	**(*Z*)-/(*E*)-β-Ocimene**	
	Linalool	
	Geraniol	
(*E,E*)-Farnesyl diphosphate	**(*E*)-β-Farnesene**	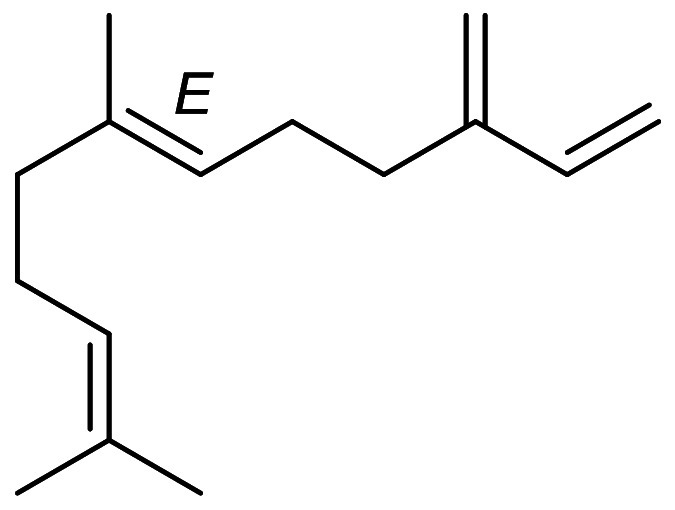
	β-Bisabolene	
	(*Z*)-γ-Bisabolene	
	(*E*)-α-Bisabolene	
(*Z,E*)-Farnesyl diphosphate	β-Bisabolene	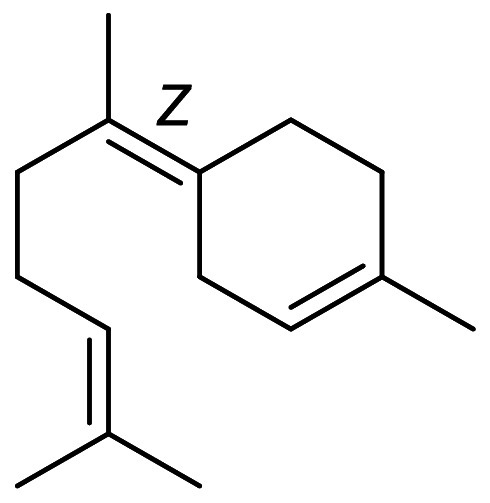
	(*Z*)-α-Bisabolene	
	**(*Z*)-γ-Bisabolene**	
	(*E*)-α-Bisabolene	
(*Z,Z*)-Farnesyl diphosphate	**β-Bisabolene**	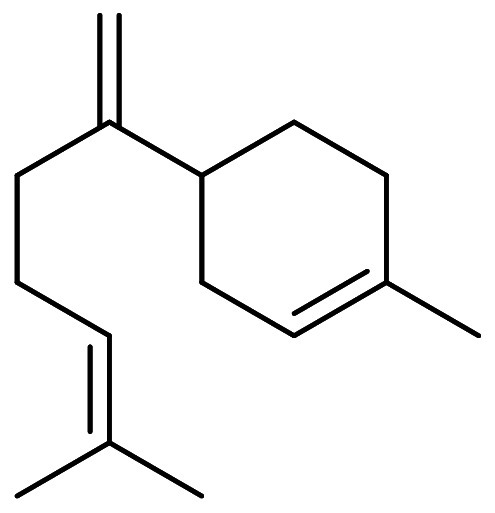
	Helminthogermacrene?	
	(*E*)-α-Bisabolene	
	(*E*)-γ-Bisabolene	
(*E,E,E*)-Geranylgeranyl diphosphate	Diterpene 1	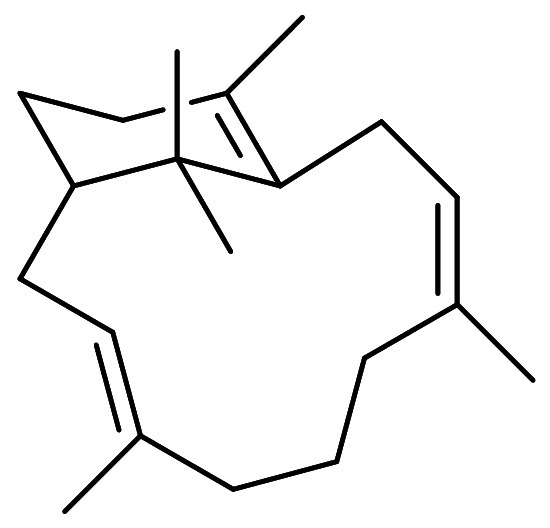
	Cembrene A	
	**Sobralene**	
	Verticillene-1	
	Verticillene-2	
	Diterpene 2	

As described in the introductory paragraphs, the sesquiterpene pheromones **6** and **7** from *L. longipalpis* are, in fact, homosesquiterpenes, which are likely derived from (*S*)-8-methylFPP. This homologue of FPP possesses an additional methyl branch, which corresponds to the position and stereochemistry of the branches seen in **6** and **7**. To establish whether the presence of a methyl group on C8 of (*E,E*)- or (*Z,E*)-FPP caused *Ll*TPS to produce either the methylgermacrene-B (**6**) or the methylhimachalene (**7**), instead of the bisabolenes, the (*E,E*)- and (*Z,E*)- isomers of 8-methylFPP were generated as substrates for *Ll*TPS in coupled enzyme assays. (±)-(*E,E*)-8-methylFPP was produced in situ by incubation of (±)-4-methylGPP with IPP in the presence of *Ll*FPPS, the *L. longipalpis trans*-FPP synthase (*SI Appendix*, Fig. S8), before addition of *Ll*TPS and incubation for a further 16 h. (±)-(*Z,E*)-8-methylFPP was similarly produced using the *cis*-FPPS *Ps*IDS3 in place of *Ll*FPPS before addition of *Ll*TPS. *Ps*IDS3 is a known (*Z,E*)-FPP synthase from the flea beetle *Phyllotreta striolata*. GC–MS analysis of the pentane extracts from both incubations did reveal the presence of homosesquiterpenes (*SI Appendix*, Figs. S9 and S10), but the EI spectra of these products did not match those of either (*S*)-9-methylgermacrene-B (**6**) (*SI Appendix*, Fig. S9) or (1*S*,3*S*,7*R*)-3- methyl-α-himachalene (**7**). It is likely that they are homobisabolenes, by analogy with the products from the standard isoprenyl pyrophosphates (*E,E*)- or (*Z,E*)-FPP.

### *Ll*TPS Produces the Diterpene Pheromone Sobralene from (*E,E,E*)-GGPP.

**Fig. 3.**
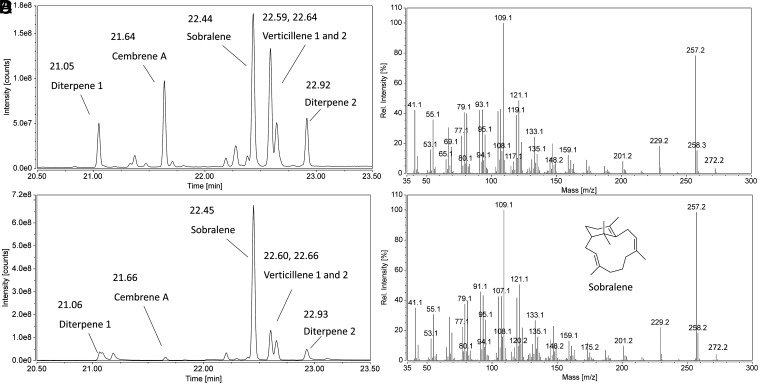
Diterpene synthase activity of *Ll*TPS. (*A*) GC–MS chromatogram of the diterpene products resulting from incubation of (*E,E,E*)-GGPP with *Ll*TPS. (*B*) GC–MS chromatogram of an extract of *L. longipalpis* males collected near Ico, Ceará state, Brazil. (*C*) EI mass spectrum of sobralene peak from chromatogram (*A*). (*D*) EI mass spectrum of authentic sobralene peak from chromatogram (*B*).

Incubation of *Ll*TPS with (*E,E,E*)-GGPP yielded a range of diterpene products (Fig. 3*A* and *SI Appendix*, Fig. S11). The major component exhibited an EI mass spectrum identical to that of sobralene (**8**), a known pheromone component of certain *L. longipalpis* chemotypes. Comparison of the GC retention time and EI mass spectrum with that seen for authentic sobralene from an extract of male *L. longipalpis* collected near Ico, Ceará state, Brazil, confirmed the identification (Fig. 3*B* and *SI Appendix*, Fig. S11). Sandflies from this region, which is approximately 400 km from Sobral, had previously been shown to produce sobralene (*SI Appendix*, Fig. S12). Other product peaks present in the GC–MS chromatogram of the *Ll*TPS assay included one with an MS very close to that of cembrene A (**9**, also known as neocembrene) and two with spectra very similar to those of verticillene isomers (**10,** **11**) (31), as well as two unknown diterpenes (*SI Appendix*, Fig. S11). These compounds were also seen as minor products in the male sandfly extract (Fig. 3*B*).

## Discussion

### *L. longipalpis* Possesses an IDS-Derived TPS.

It has long been known that insects employ a wide variety of terpenes and terpenoids as semiochemicals, whether that be as pheromones, for communication with conspecifics, or as allomones, kairomones, and synomones to mediate interspecific interactions (3). What has emerged only relatively recently is the understanding that insects are themselves capable of biosynthesizing terpenes without the direct input of plants or microorganisms. Identification of a dual function IDS/TPS in bark beetles of the genus *Ips* was the first major insight into terpene biosynthesis in insects (32), but it was the study by Beran et al. on IDSs and TPSs from the flea beetle *Phyllotreta striolata* that provided a detailed understanding of the evolutionary relationship between these two classes of enzymes (9). Males of this species of flea beetle, which feeds on the Brassicales, release *γ*-cadinene and himachalene-type sesquiterpenoids that serve as aggregation pheromones. This behavior leads to mass infestation in the field, with serious damage to commercially important crops, such as cabbage and mustard. Two active IDSs and four active TPSs were identified in *P. striolata,* and although the two enzyme types separated into two different clades, the exon–intron structures of the IDS and TPS genes were conserved, suggesting that the TPSs had evolved from the IDSs. Further evidence for this proposal was provided by Rebholz et al. in their large-scale bioinformatic analysis of insect IDS/TPS enzymes (10). Despite the small number of functionally characterized insect TPS sequences available, the authors were able to identify that disruptive amino acid substitutions in isopentenyl diphosphate–binding motifs (IBMs) were characteristic of TPSs, while IDS maintained essential consensus residues in these regions. They concluded that TPSs had emerged multiple times and independently across the insects. The identification here of *Ll*TPS from *L. longipalpis* as a TPS is consistent with these ideas, and here, we show multiple, 12 out of 15, amino acid substitutions in the IBM, when compared to the FPPS from this species, which conserved all 15 IPP-interacting residues.

### *Ll*TPS Is a Functional Terpene Synthase.

*Ll*TPS produced monoterpenes, sesquiterpenes, and diterpenes when provided with the appropriate isoprenyl diphosphate substrates in vitro. GPP is not a particularly abundant isoprenylPP metabolite in the context of most cellular environments, except when monoterpenes are actively being synthesized, and so it is likely that its role as a substrate is not biologically relevant in sandflies, which are not reported to release monoterpenes. The production of monoterpenoid alcohols by *Ll*TPS is most likely due to the small size of GPP, which can allow water molecules to enter readily the enzyme active site leading to quenching of the carbocation intermediate. Turning to sesquiterpenes, the production of (*E*)-β-farnesene from (*E,E*)-FPP, albeit with relatively low efficiency, is an interesting observation. There are no reports of this sesquiterpene as a *Lutzomyia* volatile, but it is a known feeding stimulant for the insect (33). Given this fact and the ubiquitous nature of (*E,E*)-FPP, it is quite possible that (*E*)-β-farnesene is released from the male sandfly pheromone gland, perhaps at levels below the current limit of detection.

*L. longipalpis* is a species complex, with at least eight lineages (34). Populations may be characterized into chemotypes by their pheromone chemistry. The published *L. longipalpis* genome is based on the Jacobina chemotype (27), named for that city in the Bahia state of Brazil. This population characteristically produces (1*S*,3*S*,7*R*)-3-methyl-α-himachalene (**7**) as the major component of its pheromone, together with a small amount of the regular sesquiterpene α-himachalene (18). Thus, in mining genome data of the Jacobina chemotype, it was reasonable to expect to find a TPS that produces (homo)sesquiterpenes of this structural family. Experiments performed on the *Ll*TPS reported here showed no evidence for the production of α-himachalene from the three FPP isomers used. As described above, (*E*)-β-farnesene was detected from (*E,E*)-FPP. (*Z,E*)- and (*Z,Z*)-FPP, in contrast, gave bisabolene isomers in significant quantity. These monocyclic sesquiterpenes have not been reported in any *L. longipalpis* chemotype, and we suspect that this result may be artifactual and brought about by exposure of the enzyme to uncommon (*Z,E*)- and (*Z,Z*)-isomers of FPP. That said, the flea beetle *P. striolata* possesses a *cis*-IDS and uses it to produce (*Z,E*)-FPP and from this (6*R*,7*S*)-himachala-9,11-diene (9). *Ll*TPS did not exhibit analogous activity, however. It is noteworthy that a (+)-(*S,Z*)-α-bisabolene synthase has been found in insects, namely in the green stink bug *Nezara viridula* (*Nv*TPS1 in Fig. 1*B*), but this uses (*E,E*)-FPP as a substrate to produce the bisabolene and not (*Z,E*)- or (*Z,Z*)-FPP (35, 36). The distinctly different product profiles seen between (*E,E*)- and (*Z,E*)-FPP indicate that rotation of the 2,3 bond in the carbocation intermediate is slow relative to the deprotonation step (*SI Appendix*, Fig. S13) resulting in only minor amounts of bisabolenes seen with (*E,E*)-FPP and no (*E*)-β-farnesene seen with (*Z,E*)-FPP.

Most IDSs and TPSs characterized in the literature use Mg^2+^ as a cofactor, but it has been shown that some IDSs are sensitive to changes in divalent metal ions and can switch from FPP to GPP synthase activity upon addition of Co^2+^ or Mn^2+^ (29, 30). We examined whether inclusion of either of these metals in an *Ll*TPS assay would affect the product profile, but no changes in the pattern of terpenes were seen, and Mg^2+^ was the most efficient cofactor by one to two orders of magnitude over Co^2+^ and Mn^2+^, respectively.

Given that the major pheromone component in the Jacobina chemotype is methylhimachalene **7**, a homosesquiterpene (19), we speculated that *Ll*TPS may be capable of producing this from (*E,E*)- or (*Z,E*)-8-methylFPP (*SI Appendix*, Fig. S8), even though such activity was not seen with the standard FPPs. Incubation of *Ll*TPS with either (*E,E*)- or (*Z,E*)-8-methylFPP gave homosesquiterpene products with quite different mass spectra (*SI Appendix*, Figs. S9 and 10), from those reported for the methylhimachalene **7**, or indeed the methylgermacrene **6**, demonstrating that inclusion of the additional carbon did not shift the product distribution to the known sandfly homosesquiterpenes. The male sandflies collected near Ico, Ceará state, Brazil, produced 9-methylgermacrene-B (*SI Appendix*, Fig. S10) in addition to sobralene and so we postulate that either there was a missing essential component from our assay with 8-methylFPP and *Ll*TPS, which prevented production of the methylgermacrene, or—as seems more likely—this *L. longipalpis* chemotype has an additional TPS that produces the homosesquiterpene.

### *Ll*TPS Produces the Diterpene Pheromone Sobralene from (*E,E,E*)-GGPP.

Taking the above results into account and recognizing that *Ll*TPS produces sobralene (**8**) as its major product from (*E,E,E*)-GGPP leads us to the proposal that the Jacobina chemotype harbors a gene for synthesizing this diterpene pheromone, even though it is not known to produce the compound. The exact status of the *L. longipalpis* species complex is a matter of some debate, and—as pointed out above—recent work has shown that at least eight linages exist, which may represent different species (34). Our finding of a sobralene TPS in the Jacobina chemotype may help to inform this discussion, and in the future, we intend to identify other TPSs, including homosesquiterpene synthases, in the insect. Taking together the functional FPPS and TPS reported here with the GGPPS we previously described (37), *L. longipalpis* enzymes responsible for the biosynthesis of sobralene (**8**) from DMAPP (**1**) and IPP (**2**) are now elucidated. Gene expression analysis of TPSs in a range of chemotypes could provide more insights into how the different terpene profiles are tuned. In males of *L. longipalpis,* the pheromone is released from a specialized exocrine gland located in the fourth abdominal segment of the insect (38). It is not clear whether de novo production of the terpenes takes place in these tissues, although genes associated with the MVA pathway have been found to be transcribed there (28). In the same study, an RNA contig (numbered C94), possessing high identity to *Ll*TPS, was seen in extracts of the gland. Taking these pieces of evidence together, it is likely that the pheromone gland is, indeed, the site of terpene biosynthesis.

In comparison with mono- and sesquiterpenes, true diterpenes appear relatively rare in insects. The simple acyclic diterpene springene is found in some ants, bees, and wasps (3). Cembrene A, also known as neocembrene, (**9**) is a trail pheromone in termites of the genus *Nasutitermes* (39), is the queen pheromone of the pharaoh’s ant *Monomorium pharonis* (40), and has been identified in secretions of the ant *Monomorium chinense* (41). In addition to sobralene (**8**), other diterpenes have been found in chemotypes of the *L. longipalpis* complex, either as minor components or as major products. With the exception of taxadiene (**12**), which is present in minor amounts in the extract analyzed by Palframan et al. (20), these are still to be structurally characterized. The products of *Ll*TPS included diterpenes with EI mass spectra identical to cembrene A (**9**) and verticillene (**10,** **11**) isomers (31), as well as two unknown diterpenes.

**Fig. 4.**
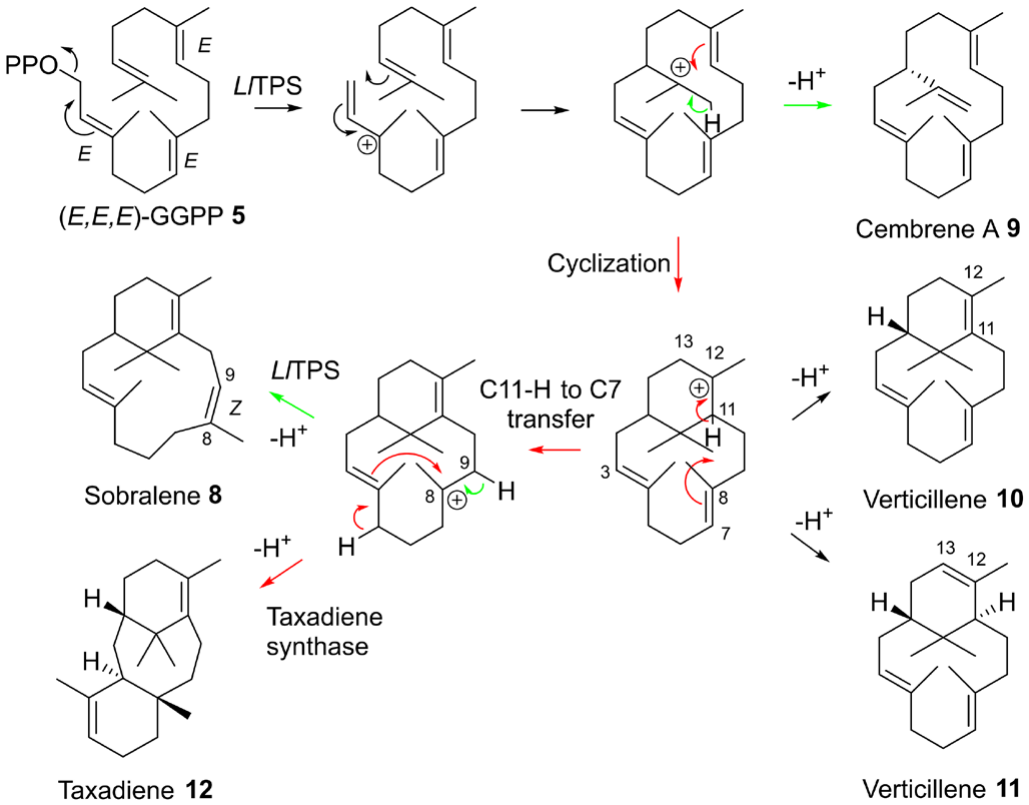
Scheme showing the proposed *Ll*TPS-catalyzed route to sobralene (**8**) from (*E,E,E*)-GGPP and its relationship with cembrene A (**9**), verticillenes (**10, 11**), and taxadiene (**12**). Alternative routes from common intermediates are shown by red or green arrows. Note that H^+^ transfer from C11 to C7 may occur directly or via C3 and that transannular ring closure and H^+^ loss to form taxadiene is a two-step process.

Intriguingly, these compounds were also present as minor components in extracts of *L. longipalpis* males found near Ico, Ceará state, Brazil; indeed, the qualitative match between the two GC chromatograms is striking (Fig. 3 *A* and *B*). Quantitatively, the pheromone extract contained proportionally more sobralene and lower amounts of the minor components than the enzyme assays. It has been shown that sobralene can be converted into the two observed verticillene isomers by treatment with acid or long-term storage of a sample of sobralene in deuterochloroform (mildly acidic conditions) (42). Thus, although the enzyme assay and sandfly pheromone extract studied here were not directly exposed to acid, it is possible that the presence of these minor compounds is an artifact of the assay conditions, sample preparation, and/or analysis. That said, enzyme assays were buffered at pH 7.2 and analyzed by GC–MS immediately following incubation. Moreover, passing the pentane fraction through a small silica gel column prior to analysis or reducing the GC injection port temperature from 200 °C to 150 °C had no effect on the quantity of minor diterpene products seen. Examination of the probable mechanism of (*E,E,E*)-GGPP cyclization shows the relationship between the diterpene components seen (Fig. 4), which is broadly in line with the proposals of Palframan and Pattenden (43). Following loss of the diphosphate group, the initial allyl cation can undergo ring closure to form a macrocycle. Loss of a proton may then lead to formation of an isopentenyl group and the product cembrene A (**9**). Alternatively, a second ring closure can occur to give the veticillenyl cation. Direct proton loss from this intermediate leads to the verticillenes (**10,** **11**), while proton migration from C11 to C7 (either directly or indirectly, via C3) followed by proton loss from C9 yields sobralene (**8**). Interestingly, taxadiene (**12**) was not detected as a product from *Ll*TPS, despite its close relationship with sobralene (**8**). This suggests that in the *Ll*TPS active site, adoption of the sobralene-like conformation of the intermediate carbocation, and subsequent proton elimination to install the *Z*-8,9 double bond of **8**, is preferred over the taxadiene-like conformation and its accompanying ring closure, following the arguments presented by Hayes et al. (44).

It is pleasing to confirm that insects are capable of making diterpenes without reliance upon the enzymatic machinery of plants or microorganisms. Currently, there is no synthetic chemical route to sobralene (**8**), and it is unlikely that sustainable and commercially viable production will be achieved by this means. Having identified a sobralene-producing enzyme, we are now exploring its potential as a viable source of the pheromone for vector control.

## Materials and Methods

### Phylogenetic Analyses.

FPPS-like homologues were identified from the *L*. *longipalpis* genome (Jacobina, NCBI accession PRJNA20279) using blastp searches with TPS enzymes from *P. striolata* as the query. Hits were assessed for the presence of IPP-binding motifs (IBMs) indicative of IDS activity, leading to the characterization of XP_055677521.1 as a likely FPPS (*Ll*FPPS) and XP_055691875.1 as a likely TPS (*Ll*TPS). These sequences were aligned with various functionally characterized insect IDS and TPS enzymes using MUSCLE. MEGA11 was used to construct the maximum likelihood tree (LG + G+I), and phylogeny was tested by bootstrap analysis (1,000 replicates). Sequences were as follows: *Dm*FPPS (NP_477380.1), *Dm*GGPPS (AAC05273.1), *Ll*GGPPS (XP_055688734.1), *Aa*FPPS (XP_001663796.1), *Ps*IDS1 (ALL35400.1), *Ps*IDS3 (ALL35406.1), *Ps*TPS1 (ALL35411.1), *Ps*TPS2 (ALL35414.1), *Ps*TPS3 (ALL35417.1), *Ps*TPS4 (ALL35420.1), *Ip*GPPS/TPS (Q58GE8.1), *Nz*TPS1 (CAH1405470.1), *Nz*TPS2 (WBV93233.1), *Mh*TPS (A0A343W969.1), *Hh*TPS1 (QBK50746.1), *Hh*TPS2 (QBA82488.1), *Mo*TPS (A0A7D0AGU9.1), *Hm*TPS1 (HmelOS), and *Hm*TPS2 (HMEL037108g1).

### Protein Expression and Purification.

Assessment of protein sequences for N-terminal localization sequences [TargetP-2.0 (45) and iPSORT (46)] revealed that *Ll*FPPS was predicted to contain mitochondrial targeting motifs. Constructs encoding full-length *Ll*TPS (aa 1 to 340) and truncated *Ll*FPPS (aa 72 to 411) for recombinant protein expression in bacteria (pET100/D-TOPO, N-terminally His-tagged) were synthesized by GeneArt (Thermo Fisher Scientific, Loughborough, UK). These were transformed into BL21(DE3)pLysS *E. coli,* and 20 mL LB cultures were grown from a single colony (100 µg/mL ampicillin and 34 µg/mL chloramphenicol). Following 16 h at 37 °C with shaking (200 rpm), these were added to 1 L media and incubated at 37 °C until reaching OD_600_ 0.5, at which point IPTG was added to 0.75 mM and cultures were incubated at either 25 °C for 5 h (*Ll*TPS) or 37 °C for 3 h (*Ll*FPPS). Cells were harvested by centrifugation (4,000 rpm, 15 min, 4 °C) and stored at −80 °C.

For purification, cell pellets were lysed in 30 mL wash buffer (50 mM Tris-HCl pH 7.4, 100 mM NaCl, 10 % v/v glycerol, 20 mM imidazole, and 1 mM DTT) supplemented with 1 v/v Igepal CA-630 and EDTA-free cOmplete protease inhibitor (Roche) and incubated at 4 °C for 15 min. Cell extracts were then sonicated (12 × 10 s on ice) and the insoluble fraction pelleted by centrifugation (14,000 rpm, 20 min, 4 °C). Nickel-NTA agarose beads (0.5 mL bed) were added to the soluble fraction and rotated (90 min, 4 °C), before washing (3 × 10 mL wash buffer) and elution (3 mL wash buffer plus 400 mM imidazole). Proteins were buffer exchanged into protein storage buffer (25 mM MOPSO pH 7.2, 100 mM NaCl, 10% v/v glycerol, and 1 mM DTT) using PD-10 columns (Cytiva), with aliquots snap-frozen (N_2(l)_) and stored at −80 °C. Expression and purification of *Ps*IDS3 (aa 31 to 384) was performed with the same methodology (37 °C for 3 h postinduction).

### Protein Mass Spectrometry.

For assessment of tryptic peptides, protein aliquots (*Ll*TPS and *Ll*FPPS) were denatured with 5× Laemmli SDS sample buffer (Alfa Aesar, Thermo Fisher Scientific) and incubation at 95 °C for 5 min. Protein was loaded onto a 12% tris-glycine gel (BioRad) and resolved by SDS-PAGE (160 V, 400 mA, 50 min). The gel was stained with GelCode™ Blue Safe Protein Stain (Thermo Fisher Scientific) as per the manufacturer’s instructions. Bands corresponding to the target proteins were excised, reduced (10 mM dithiothreitol), and alkylated (55 mM iodoacetamide) followed by addition of aqueous trypsin (0.5 μg, Promega) and incubation at 37 °C for 18 h. Digestion was quenched by the addition of formic acid (1 μL, Honeywell, Fisher Scientific), and the supernatant was centrifuged and subjected to high-performance liquid chromatography–tandem mass spectrometry (LC–MS/MS) analysis.

Digests were analyzed using an Ultimate 3000 RSLCnano HPLC system fitted with a PepMap 300 C18 column (15 cm × 150 μm, 5 μm particle size, Thermo Fisher Scientific) coupled to a Thermo LTQ Ultra Hybrid mass spectrometer (linear ion trap/FTICR MS) via a nano-ESI source equipped with a fused silica emitter tip (20 μm ID, MSWIL, Aarle-Rixtel, The Netherlands). A dual mobile phase gradient was used composed of 5:95:0.1 (A) and 95:5:0.1 (B) water:acetonitrile:formic acid with a flow rate of 400 μL per minute. MS analysis was performed in data-dependent acquisition mode whereby the three most abundant ions in the full scan were subjected to collision-induced dissociation fragmentation. MS/MS data were analyzed using SearchGUI. In brief, RAW files were first converted to the compatible MZ5 format using MSConvert (47) and then subjected to a search using X!Tandem (48) against a UniProt database (1,117,184 total sequences) followed by visualization in Peptide Shaker (49). Identified peptides were mapped to the protein sequence (*SI Appendix*, Fig. S2 *D* and *E*) using MS Tools (50).

For assessment of protein native mass, *Ll*TPS protein was concentrated approximately twofold using a Vivaspin centrifuge column (30 K MWCO, Sartorius) and buffer exchanged into 50 mM ammonium acetate. Native mass spectrometry analysis was performed on a Waters Synapt G1 Quadrupole Time-of-Flight MS operating in positive ion mode. Samples were sprayed from pulled glass emitter tips (0.3 μm internal diameter) back filled with platinum wire. A 1.1 kV potential was applied to the tip with 0.1 mL per minute nitrogen for desolvation and minimal energies (25 V) applied to the trap and transfer cells.

### IDS Activity Assays.

IDS activity was assessed by combining recombinant protein (2 µM) with allylic substrate (50 µM DMAPP/100 µM 4-methylGPP) and 100 µM IPP in assay buffer (25 mM MOPSO pH 7.2, 10 mM MgCl_2_, and final volume 100 µL) and incubating for 1 h at 30 °C. DMAPP (38426) was purchased from Merck (Gillingham, UK) and IPP (I-0050) from Echelon Biosciences.

For assessment by reverse-phase LC–MS, 20 mM sodium diphosphate was added to samples, and 5 µL was injected onto a Waters Acquity Ultra Performance Liquid Chromatography system with a BEH C18 column (1.7 μM, 2.1 mm × 50 mm; Waters Corporation) coupled to a Waters Synapt G1 Quadrupole Time-of-Flight MS operating in negative electrospray ionization mode. The mobile phase flow rate was maintained at 0.3 mL/min, consisting of a binary gradient of 5 mM ammonium bicarbonate (solvent A) and acetonitrile (solvent B). Gradient parameters were as follows: starting at 10% v/v B, increasing to 65% B over 11.00 min, then to 95% B by 11.10 min (0.9-min hold time to 12.00 min), before returning to 10% B by 12.10 min (held until 14.00 min). Capillary voltage was −2.3 kV, with a desolvation temperature of 500 °C and gas flow rate of 600 L/h, and FPP was identified by its deprotonated ion (EIC *m/z* 381.2).

To analyze products by gas chromatography–mass spectrometry (GC–MS), enzyme assays were set up and incubated as described above, followed by addition of 20 U shrimp alkaline phosphatase (SAP, New England Biolabs) and incubation for 4 h at 37 °C. Cyclohexane (200 µL) was added and assays were left overnight, before mixing the aqueous and organic layers, centrifuging (3,000 × g for 2 min) and discarding the aqueous layer. The organic layer was evaporated to ~50 µL under a stream of nitrogen, and 5 µL was injected onto an Agilent-Jeol GC–MS (see below for details). Product identities were confirmed by comparison with MS spectra and retention times of known standards.

### TPS Activity Assays.

TPS activity was assessed by combining 2 µM protein with allylic substrate [50 µM GPP, (*E,E*)-FPP, (*Z,E*)-FPP, (*Z,Z*)-FPP, or (*E,E,E*)-GGPP] in assay buffer (final volume 100 µL, 10 mM MgCl_2_ was replaced with an equal concentration of either MnCl_2_ or CoCl_2_ in experiments designed to test the efficacy of those ions), addition of 200 µL pentane, and incubation at 30 °C overnight. GPP (G6772), (*E,E*)-FPP (44722), and *(E,E,E*)-GGPP (G6025) were purchased from Merck and (*Z,E*)-FPP (I-0180) and (*Z,Z*)-FPP (I-0170) from Echelon Biosciences. The aqueous and organic layers were mixed with a pipette, separated by centrifugation (3,000 × g for 2 min), and the aqueous layer discarded. The organic layer was evaporated to ~50 µL under a stream of nitrogen and assessed by GC–MS using either the Agilent-Jeol GC–MS or the Thermo ISQ 7000 GC–MS (see below for details). Product identities were confirmed by comparison with MS spectra and retention times of known standard compounds or pheromone extracts, where available. Where identification relied upon library spectrum fit only (i.e., helminthogermacrene and verticillenes-1 and -2), it is listed as tentative. IDS-TPS coupled assays were carried out similarly, but with incubation of 2 µM IDS protein with 100 µM 4-methylGPP and 100 µM IPP for 1 h at 30 °C, before addition of 2 µM TPS enzyme and further incubation overnight overlaid with pentane.

### Synthesis of (±)-4-methylgeranyl Diphosphate.

(±)-4-methylgeraniol was purchased from Enamine. ^1^H NMR (400 MHz, CDCl_3_) δ 5.44 (t, ^3^*J*_H,H_ = 6.8 Hz, 1H), δ 5.07 (t, ^3^*J*_H,H_ = 7.2 Hz, 1H), 4.19 (d, ^3^*J*_H,H_ = 6.7 Hz, 2H), 2.14 (m, 1H), 2.05 (m, 2H), 1.71 (s, 3H), 1.64 (s, 3H), 1.62 (s, 3H), 1.01 (d, ^3^*J*_H,H_ = 6.6 Hz, 3H); ^13^C NMR (101 MHz, CDCl_3_) δ 144.0, 132.0, 123.6, 123.1, 59.5, 42.9, 33.5, 25.8, 19.0, 17.8, 13.4 (*SI Appendix*, Fig. S14). (±)-4-methylgeranyl diphosphate was synthesized from the alcohol via the tosylate following a literature method (51) to yield 20 mg of product. ^1^H NMR (500 MHz, D_2_O) δ 5.38 (t, ^3^*J*_H,H_ = 6.7 Hz, 1H), δ 5.09 (t, ^3^*J*_H,H_ = 7.1 Hz, 1H), 4.40 (m, 2H), 2.13 (q, 7.0 Hz, 1H), 2.01 (m, 2H), 1.60 (s, 3H), 1.68 (s, 3H), 1.53 (s, 3H), 0.92 (d, ^3^*J*_H,H_ = 6.8 Hz, 3H); ^13^C NMR (125 MHz, D_2_O) δ 142.3, 133.9, 123.3, 119.3, 62.5, 42.4, 32.8, 24.9, 18.4, 17.1, 12.7 (*SI Appendix*, Fig. S15); ^31^P NMR (162 MHz, D_2_O) δ −6.74 (d, ^2^*J*_P,P_ = 21.7 Hz), −10.43 (d, ^2^*J*_P,P_ = 21.3 Hz) (*SI Appendix*, Fig. S16).

### Gas Chromatography–Mass Spectrometry.

GC–MS was performed on either an Agilent 7890B GC equipped with a DB-5 ms column (30 m × 0.25 mm × 0.25 µm, Agilent) coupled to a Jeol AccuTOF GCx system, or a Thermo ISQ 7000 GC–MS system equipped with a Trace Gold TG17-MS column (30 m × 0.25 mm × 0.25 µM, Thermo Fisher Scientific). For both instruments, the inlet temperature was set to either 150 °C or 200 °C, operated in splitless mode. For the Agilent-Jeol GC–MS, the GC oven temperature was held at 35 °C for 4 min, then increased at 10 °C/min to 300 °C followed by a 6-min hold time. The helium carrier gas flow rate was 1 mL/min. The MS was operated in positive-ion electron ionization mode, and the mass range scanned from m/z 40 to 500. For the Themo GC–MS, the oven program started with a 3-min isothermal hold at 35 °C, followed by an increase of 10 °C/min to 260 °C and a 5-min hold. The helium carrier gas flow rate was 1.5 mL/min. The MS was operated in positive electron ionization mode, and the mass range scanned from m/z 40 to 450. Both mass spectrometers operated with an 8-min solvent delay, which was reduced to 5 min for monoterpene analysis. Kovats retention indices were estimated using an alkane C_8_-C_20_ standard (Merck). Authentic standards/extracts were as follows: (*E*)-β-farnesene (provided by Pickett), geraniol (Merck), bisabolene (mix of isomers, Thermo Fisher Scientific), cedarwood Himalayan oil (himachalenes, Nikura), and *L. longipalpis* pheromone extract [(*S*)-9-methylgermacrene-B, sobralene, see below].

### Insect Collection.

*L. longipalpis* flies were collected in the Alfa settlement (latitude −6.424452570; longitude −38.9214688), near Ico, Ceará, Brazil, during the period from 30.05.2023 to 01.06.2023. Traps, of the CDC type, were placed near farms with chickens and goats from 5:00 PM to 6:00 AM each night. In total, 577 sandfly specimens were captured (381 males and 196 females). Collected insects were placed in vials containing hexane (HPLC grade, Merck) using 2 mL of solvent for males and 1 mL for females. Both samples were filtered, and the hexane solution was stored at −20 °C.

## Supplementary Material

Appendix 01 (PDF)

## Data Availability

Mass spectrometry data created during this research are openly available from the University of Nottingham data repository at https://doi.org/10.17639/nott.7364 (52). All other data are included in the manuscript and/or *SI Appendix*.
