# Supplementary material for: A diterpene synthase from the sandfly Lutzomyia longipalpis produces the pheromone sobralene
Source: Proc Natl Acad Sci U S A. 2024 Mar 12;121(12):e2322453121. doi: 10.1073/pnas.2322453121 (PMC10962984; doi:10.1073/pnas.2322453121)
Supplement: Supplementary file 1 — Appendix 01 (PDF) [file pnas.2322453121.sapp.pdf]

PNAS

Supporting Information for
A novel diterpene synthase from the sandfly *Lutzomyia longipalpis* produces the pheromone sobralene.

Charles Ducker, Cameron Baines, Jennifer Guy, Antônio Euzébio Goulart Santana, John A. Pickett*, Neil J. Oldham*

Neil J. Oldham
Email: neil.oldham@nottingham.ac.uk

John A. Pickett
Email: pickettj4@cardiff.ac.uk

This PDF file includes:

Figures S1 to S16
Tables S1

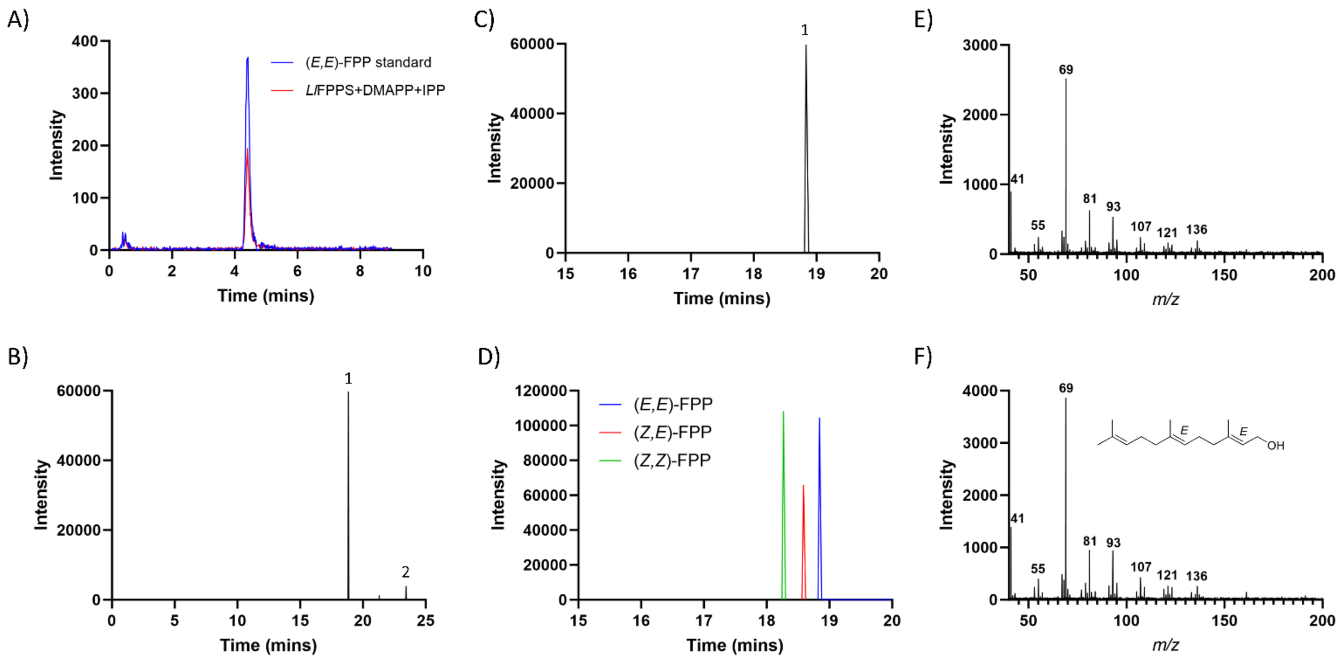

Figure S1. *Ll*XP_055677521.1 is an active FPPS. A) Overlay of LC-ESI-MS extracted ion chromatogram (*m/z* 381.2) of the product from incubation of *Ll*FPPS (5 µM) + DMAPP (50 µM) + IPP (100 µM), together with that of 10 µM (*E,E*)-FPP standard. B) GC-MS chromatogram of products from incubation of *Ll*FPPS (2 µM) with DMAPP (50 µM) and IPP (100 µM), followed by treatment with shrimp alkaline phosphatase (SAP, 20 U) to yield the free alcohol, extracted into cyclohexane. 1 = farnesol (from FPP), 2 = geranylgeraniol (from GGPP). C) GC-MS chromatogram expansion from B) highlighting product 1. D) Overlaid GC-MS chromatograms of farnesol isomers following treatment of 50 µM (*E,E*), (*Z,E*) or (*Z,Z*)-FPP standards with SAP (20 U), extracted into cyclohexane. E) EI mass spectrum of product 1 from chromatograms B)/C). F) EI mass spectrum of authentic (*E,E*)-farnesol from D).

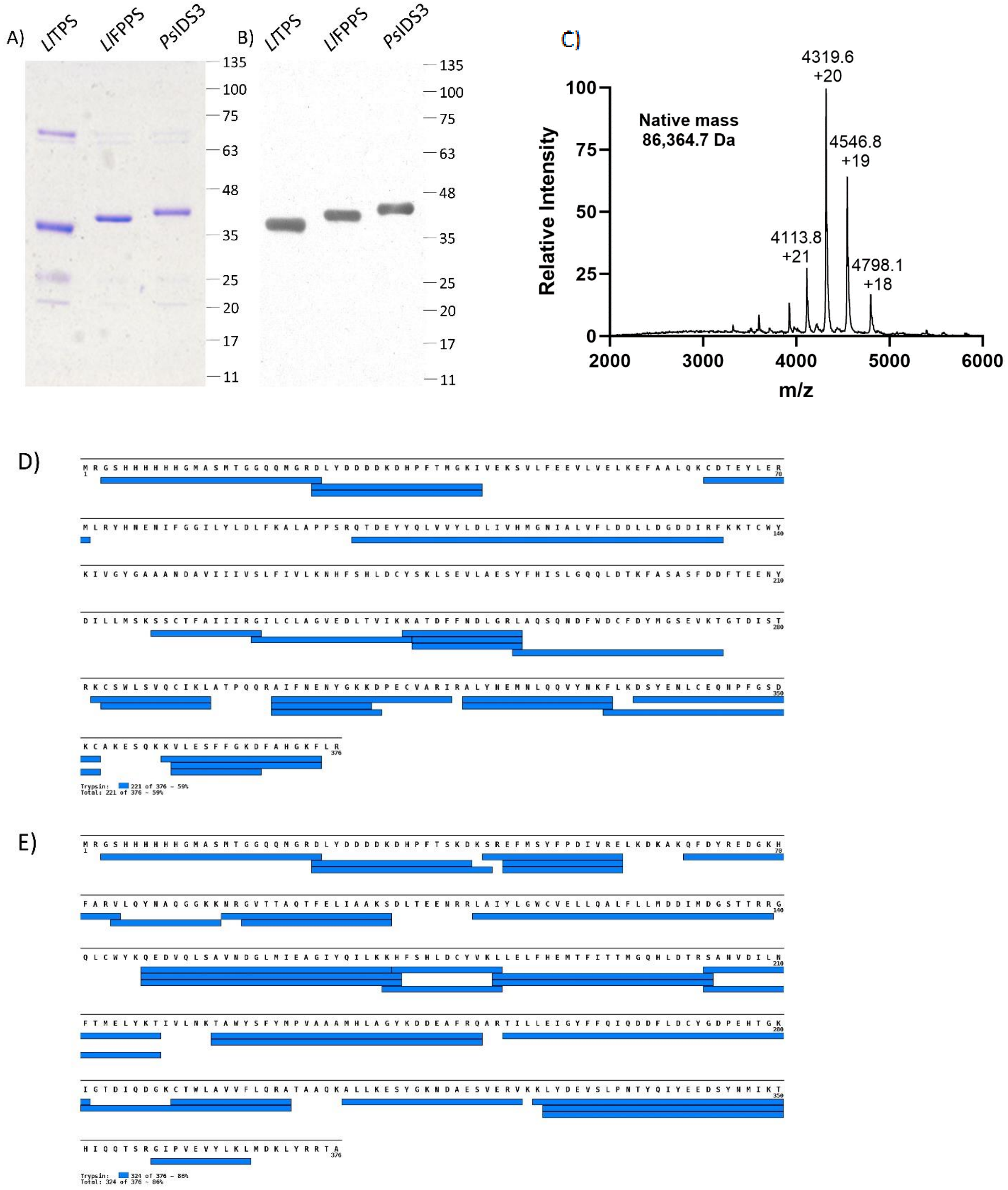

Fig. S2. Characterization of *Ll*TPS. (A) Equal loads (1.5 µg) of recombinant *Ll*TPS, *Ll*FPPS and *Ps*IDS3 resolved by SDS-PAGE (5-20% gradient) and stained using Coomassie Blue R-250. Numbers to right denote MW markers (kDa). (B) Gel from (A) transferred to nitrocellulose membrane and immunoblotted using αHis-tag antibody (Bio-Rad AD1.1.10). (C) Native electrospray (ESI) mass spectrum showing the dimeric structure of *Ll*TPS. (D) MS/MS coverage following tryptic digestion of recombinant *Ll*TPS (59% coverage). (E) MS/MS coverage following tryptic digestion of recombinant *Ll*FPPS (86% coverage). Blue bars represent regions of sequence with positive identifications.

Table S1. Terpene and terpenoid products of *Ll*TPS. Literature measurements taken from The Pherobase (DB-5).

Name	Kovat's Index (5% diphenyl 95% dimethyl polysiloxane)			Kovat's Index (50% diphenyl 50% dimethyl polysiloxane)	
	Enzyme product	Standard	Literature	Enzyme product	Standard
Monoterpenes					
Geraniol	1262	1262	1255	1438	-
Linalool	1103	-	1098	1201	-
β-Myrcene	-[a]	-	-	1000	-
Limonene	-[a]	-	-	1058	-
(*Z*)- β-Ocimene	1028	-	1040	1085	-
(*E*)- β-Ocimene	-[a]	-	-	1097	-
Sesquiterpenes					
(*E*)-β-Farnesene	1457	1457	1458	1580	1579
β-Bisabolene	1515	1515	1509	1643	1643
(*Z*)-α-Bisabolene	1508	1507	1504	1647	1647
(*Z*)-γ-Bisabolene	1519	1519	1515	1668	1668
Helminthogermacrene?	1508	-	-	1668	-
(*E*)-γ-Bisabolene	1535	1535	1541	1687	1686
(*E*)-α-Bisabolene	1548	1548	1549	1691	1690
Homofarnesene?	-[a]	-	-	1617	-
Homobisabolene 1?	-[a]	-	-	1664	-
Homobisabolene 2?	1546	-	-	1687	-
Homobisabolene 3?	1587	-	-	1747	-
α-Himachalene	-	1457	1447	-	1605
β- Himachalene	-	1510	1499	-	1665
γ- Himachalene	-	1487	-	-	1643
9-Methylgermacrene-B	-	1608	-	-	1775
Methylelemene	-	1511	-	-	1648
Diterpenes					
Diterpene 1	1905	-[a]	-	2056	2056
Cembrene A	1955	-[a]	-	2106	2106
Sobralene	2009	2007	-	2172	2173
Verticillene-1	2021	2017	-	2185	2185
Verticillene-2	-[a]	-[a]	-	2190	2190
Diterpene 2	2052	-[a]	-	2213	2213
Taxadiene	-	-	-	-	2189

[a]Not detected on this (lower sensitivity) instrument.

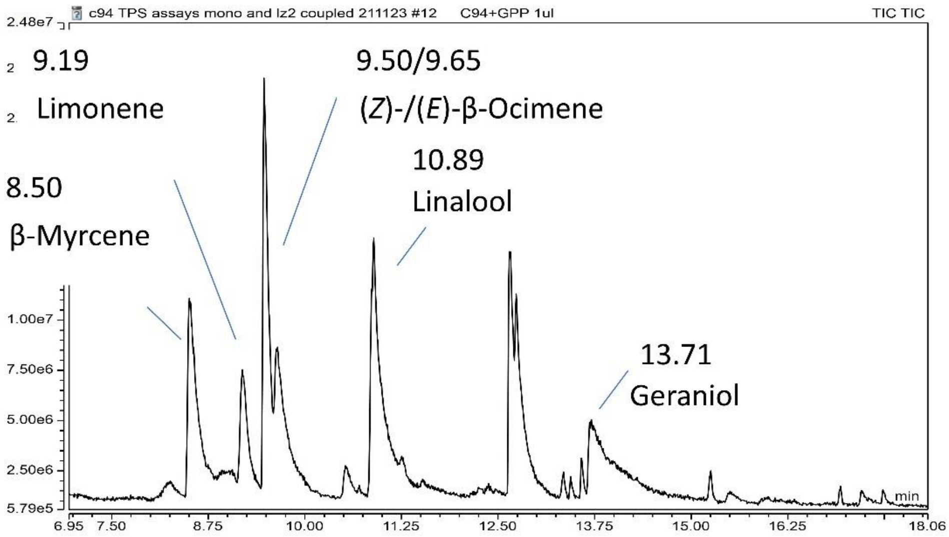

Fig. S3. GC-MS chromatogram of the products resulting from incubation of *Ll*TPS with GPP.

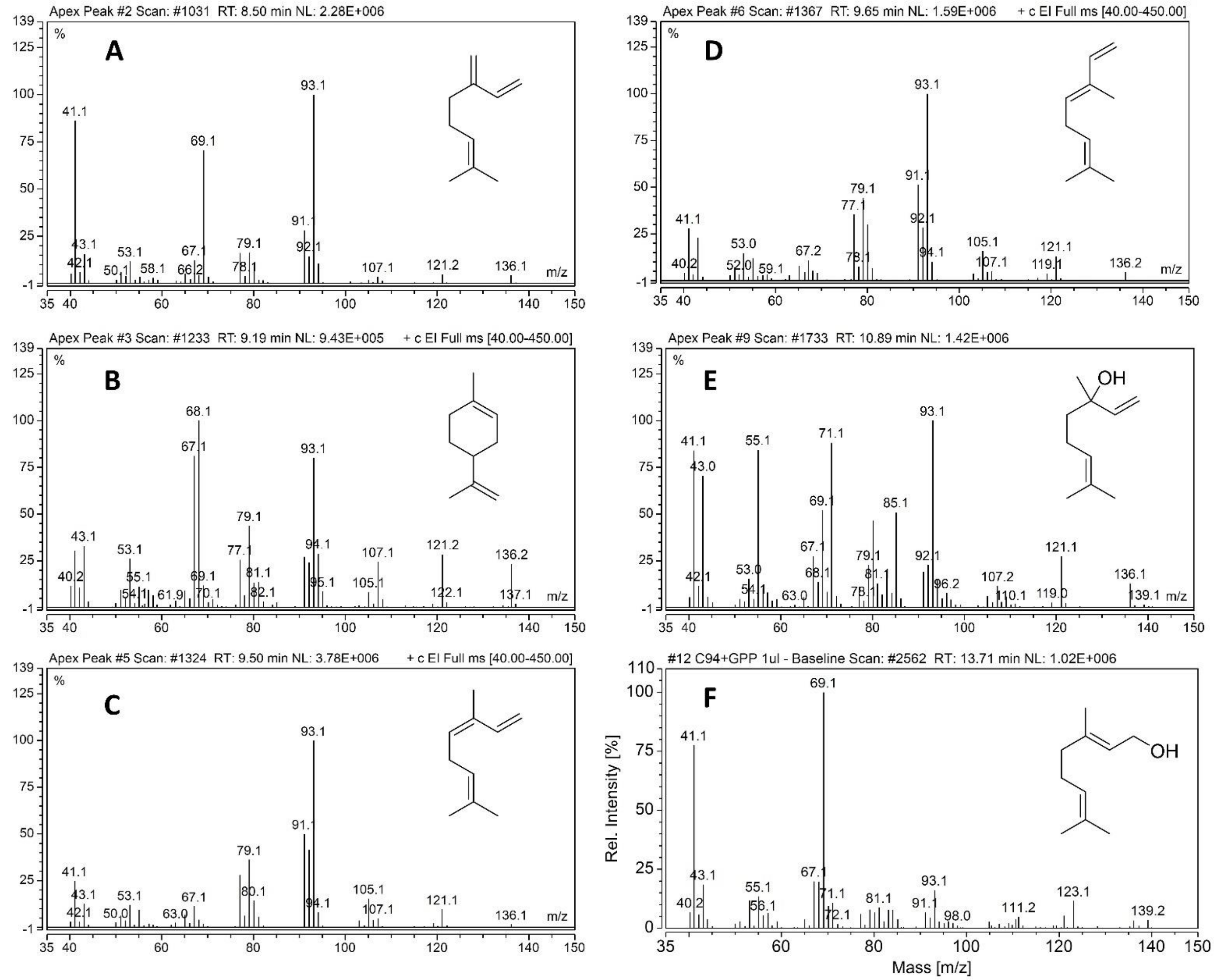

Fig. S4. EI mass spectra of the principal peaks from Fig. S2. (A) myrcene, (B) limonene, (C) (*Z*)-β-ocimene, (D) (*E*)-β-ocimene, (E) linalool, (F) geraniol.

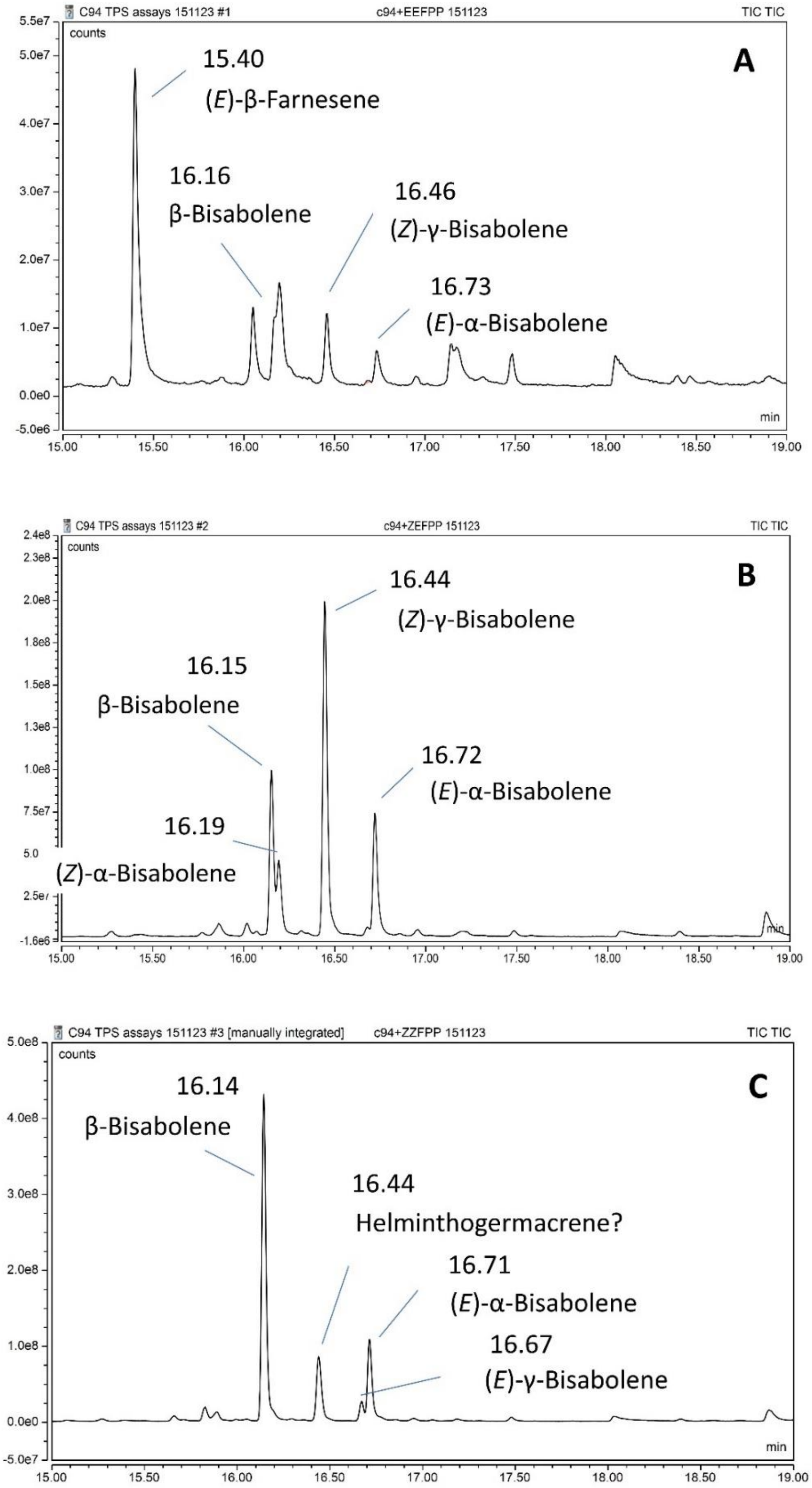

Fig. S5. GC-MS chromatograms of the products resulting from incubation of *Ll*TPS with (A) (*E*,*E*)-FPP, (B) (*Z*,*E*)-FPP, and (C) (*Z*,*Z*)-FPP.

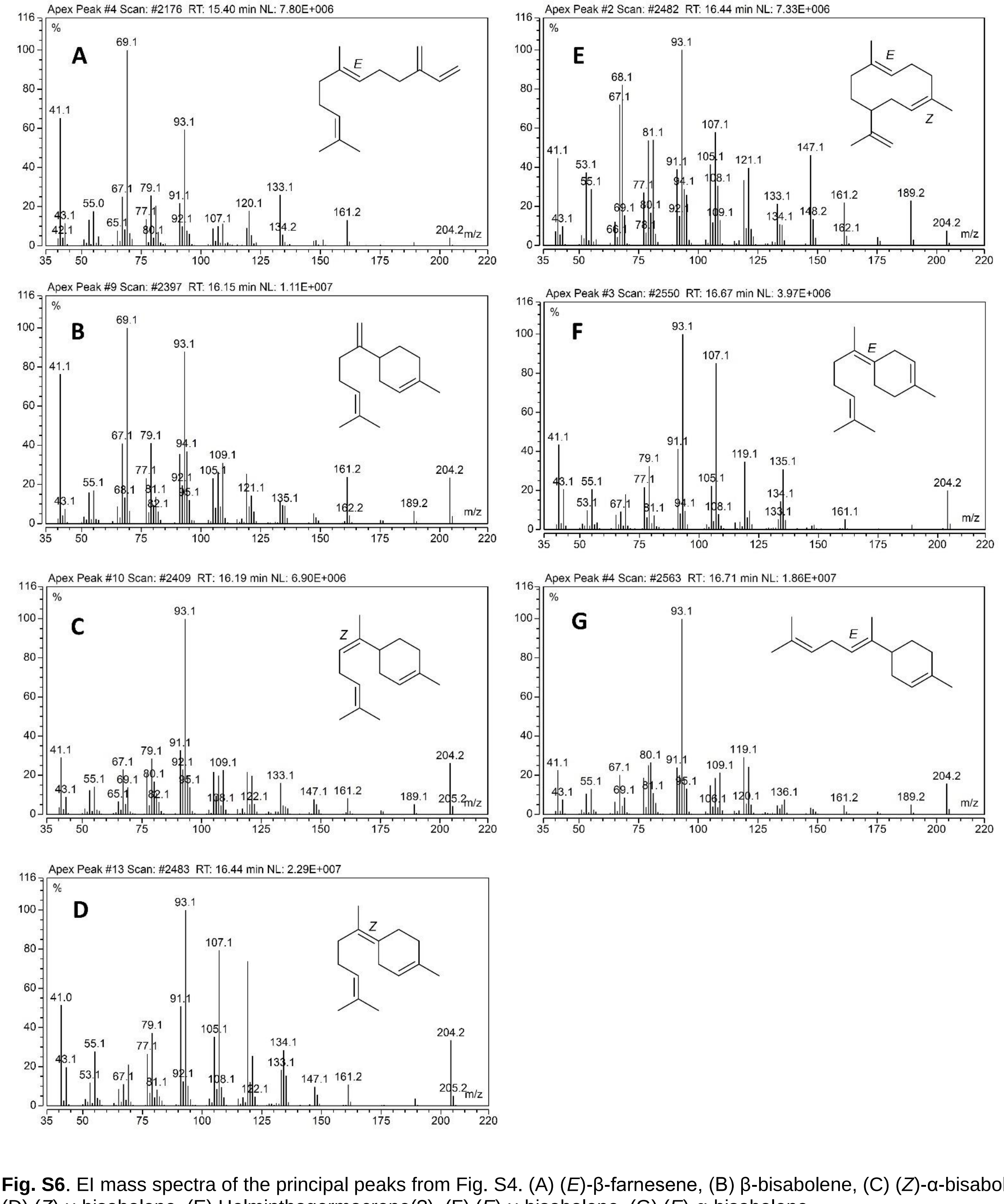

Fig. S6. EI mass spectra of the principal peaks from Fig. S4. (A) (*E*)-β-farnesene, (B) β-bisabolene, (C) (*Z*)-α-bisabolene, (D) (*Z*)-γ-bisabolene, (E) Helminthogermacrene(?), (F) (*E*)-γ-bisabolene, (G) (*E*)-α-bisabolene.

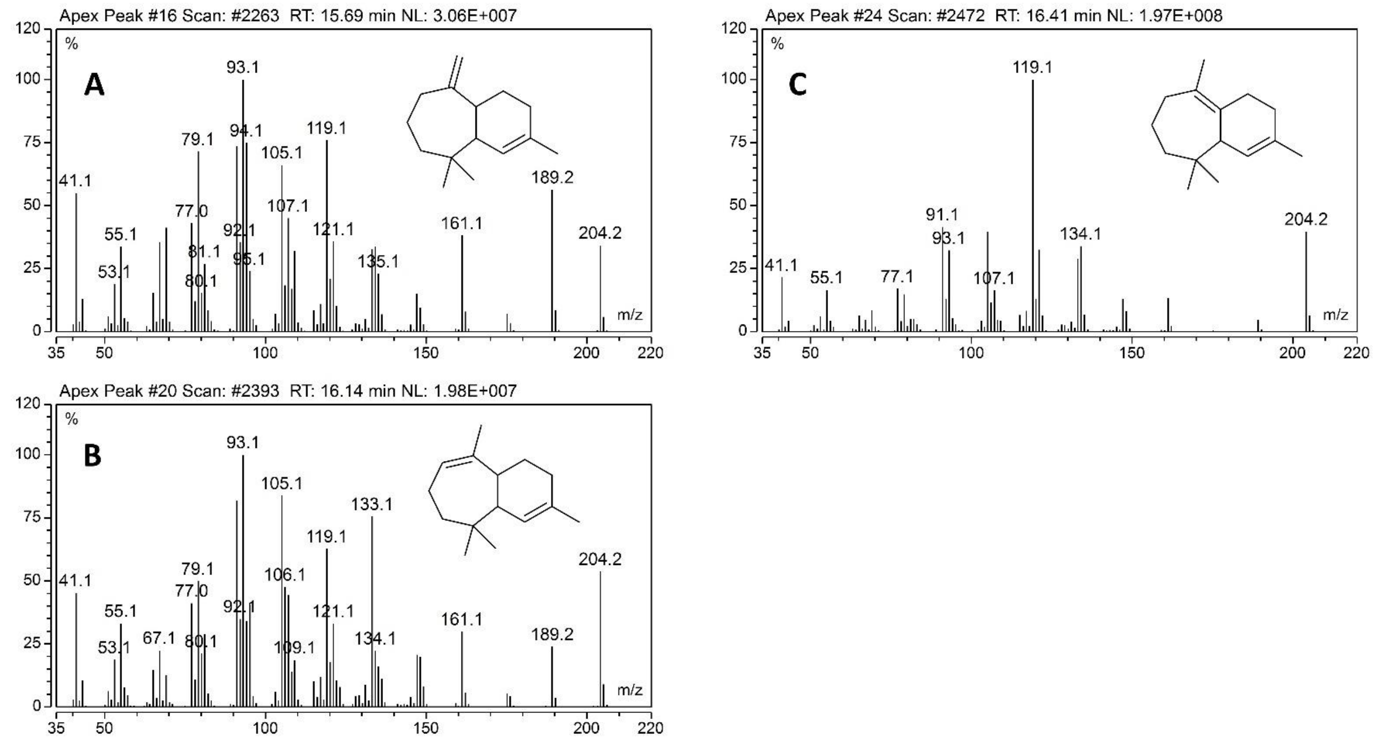

Fig. S7. EI mass spectra of himachalene standards not seen as products in *Ll*TPS assays with FPP isomers. (A) α-himachalene, (B) γ-himachalene, and (C) β-himachalene.

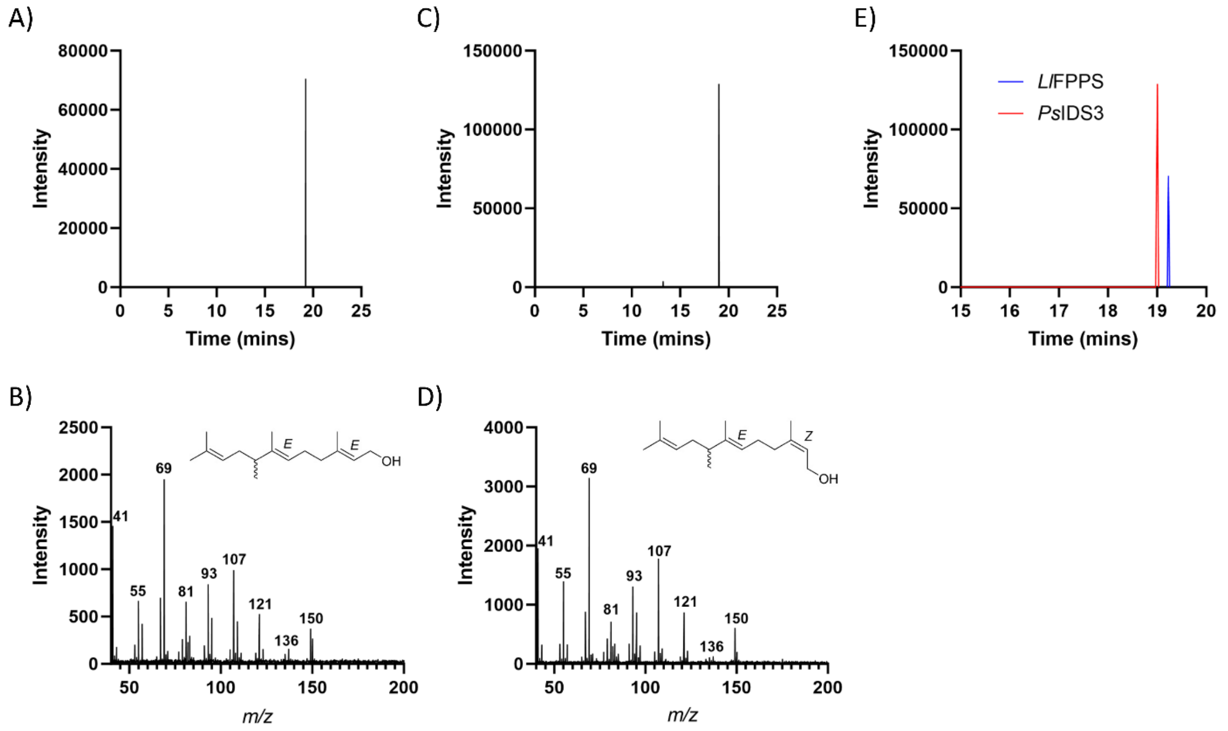

Fig. S8. Production of (±)-(*E,E*)- and (±)- (*Z,E*)-8-methylFPP by elongation of (±)-4-methylGPP with IPP and either *Ll*FPPS or *Ps*IDS3, respectively. (A) GC-MS chromatogram from incubation of *Ll*FPPS (2 µM) with (±)-4-methylGPP (100 µM) and IPP (100 µM), followed by treatment with 20U SAP and cyclohexane extraction. (B) EI mass spectrum of (±)- (*E,E*)-8-methyl farnesol from (A). (C) GC-MS chromatogram from incubation of *Ps*IDS3 (2 µM) with (±)-4-methyl GPP (100 µM) and IPP (100 µM), followed by treatment with 20 U SAP and cyclohexane extraction. (D) EI mass spectrum of (±)- (*Z,E*)-8-methyl farnesol from C). E) Overlay of GC-MS chromatograms of the two (±)-8-methyl farnesol isomers from (A) and (C).

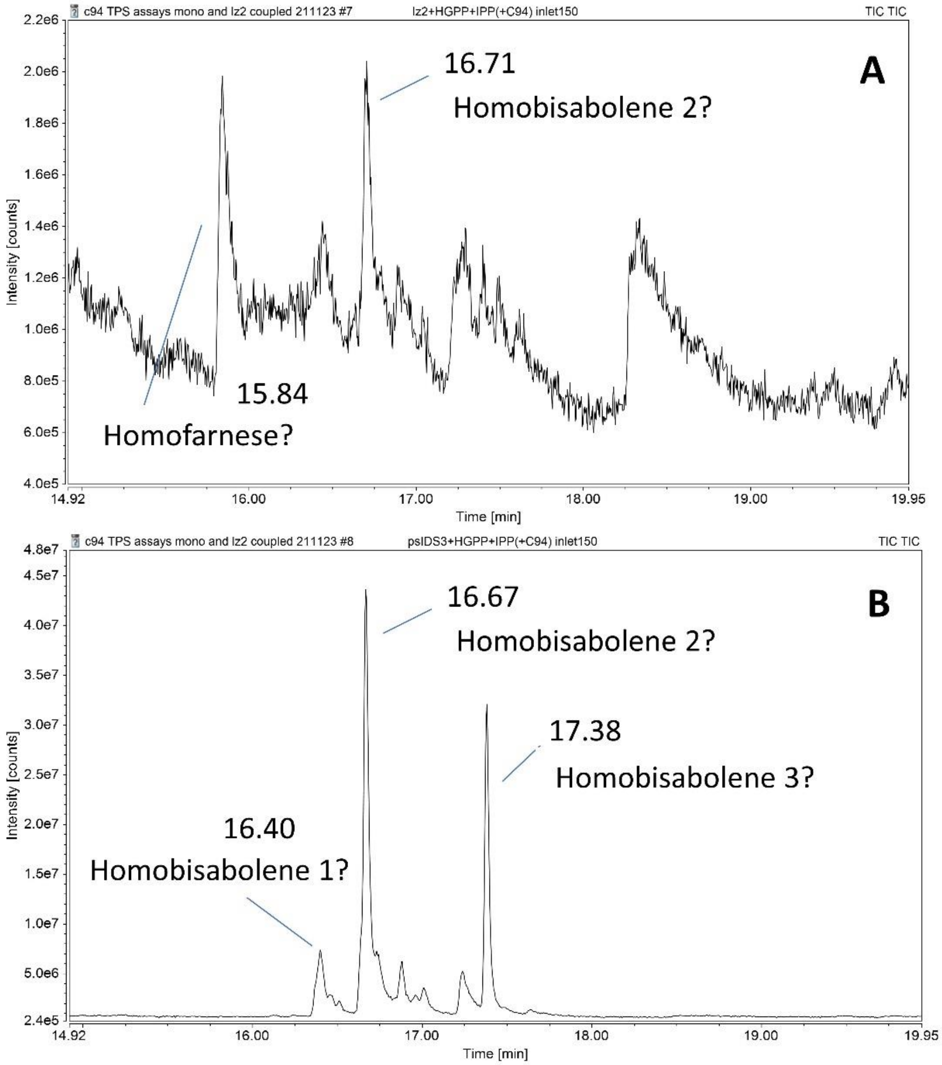

Fig. S9. GC-MS chromatograms of the products resulting from incubation of *Ll*TPS with (A) (±)-(*E*,*E*)-8-methylFPP and (B) (±)-(*Z*,*E*)-8-methylFPP.

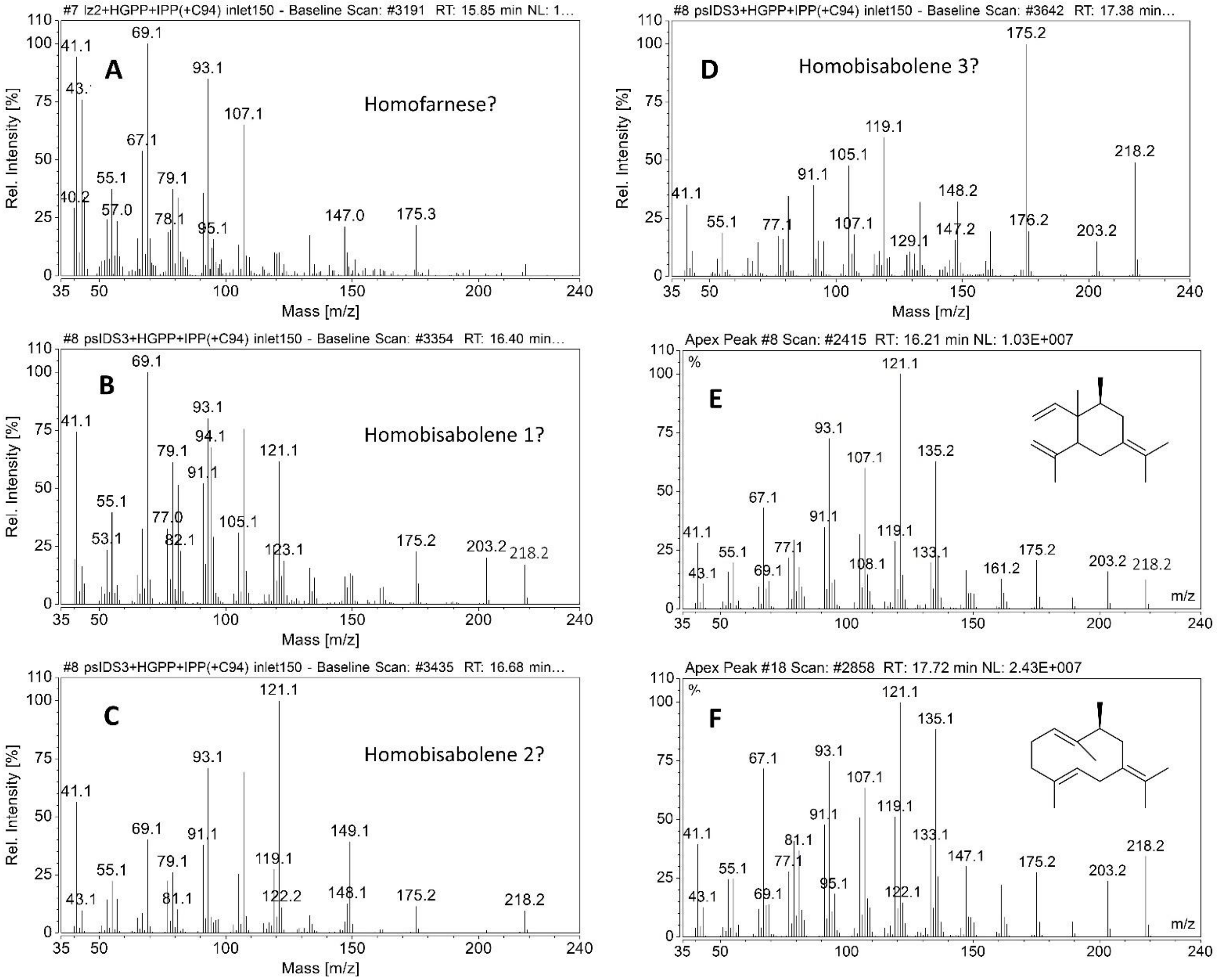

Fig. S10. EI mass spectra of homosesquiterpene products from incubation of *Ll*TPS with (±)- (*E,E*)-8-methylFPP (A, C) and (±)- (*Z,E*)-8-methylFPP (B-D). EI mass spectra of methylelemene (E) and 9-methylgermacrene-B (F) produced by *L. longipalpis* males collected near Ico, Ceará state, Brazil. Methylelemene is an artefactual thermal rearrangement product of methylgermacrene.

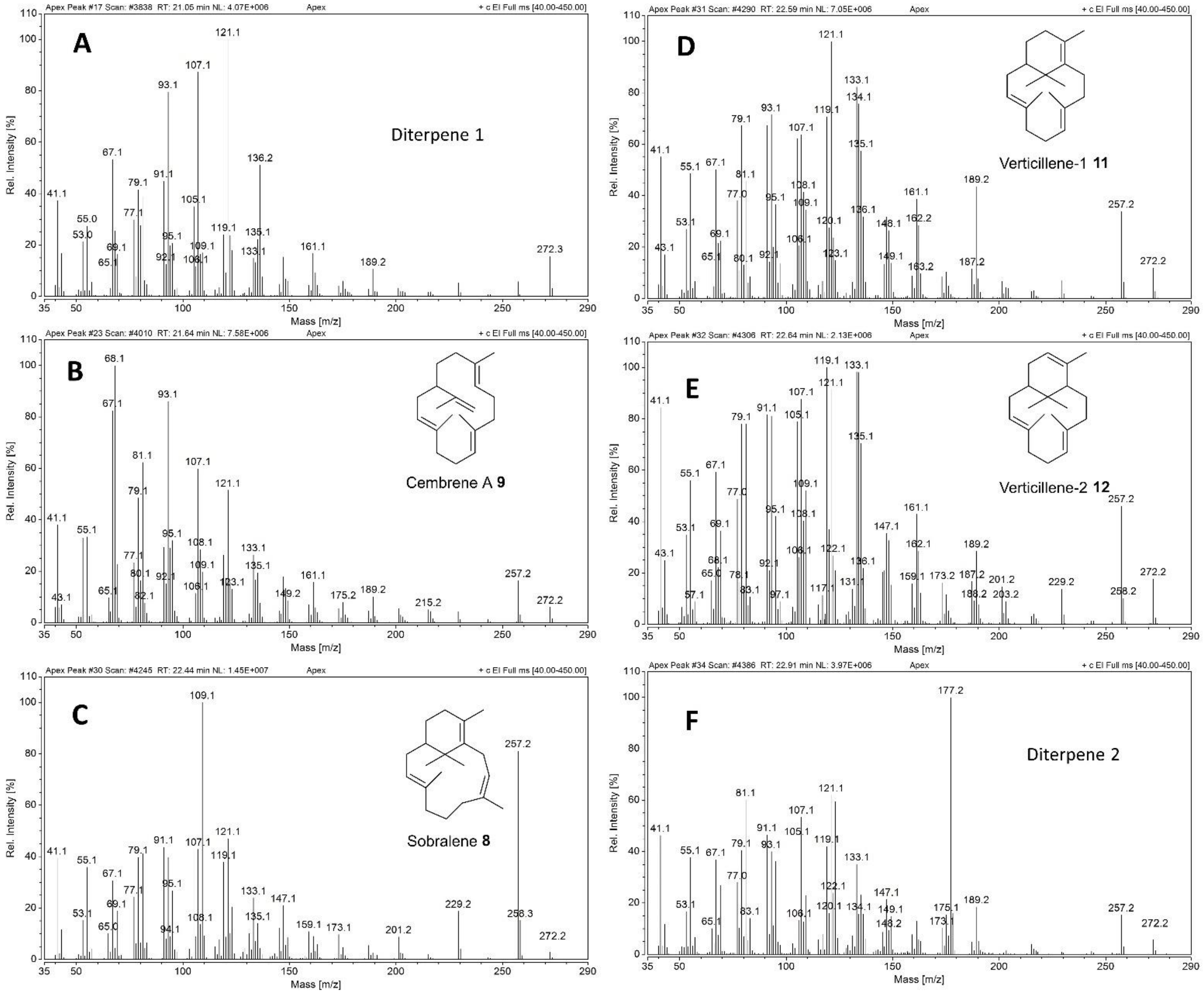

Fig. S11. EI mass spectra of diterpene products from incubation of *Ll*TPS with (*E,E,E*)-GGPP (A) unknown diterpene 1, (B) cembrene A, (C) sobralene, (D) verticillene-1?, (E) verticillene-2?, (F) unknown diterpene 2.

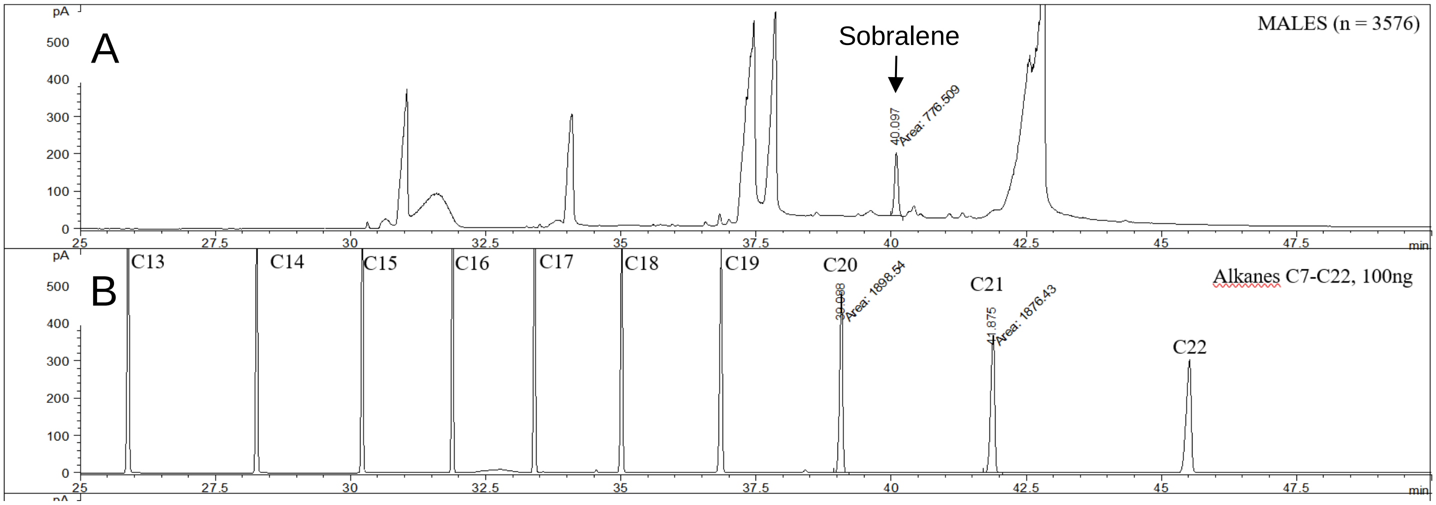

Fig. S12. GC traces of (A) an extract of *L. longipalpis* males collected in the Alfa settlement (latitude -6.424452570, longitude -38.9214688), near Ico, Ceará, Brazil, and (B) a linear alkane standard. The sobralene peak in (A) is at 40.097 min. Minor components are seen eluting just before and just after 40 min. Data collected by Hooper, Dufour, Santana and Pickett.

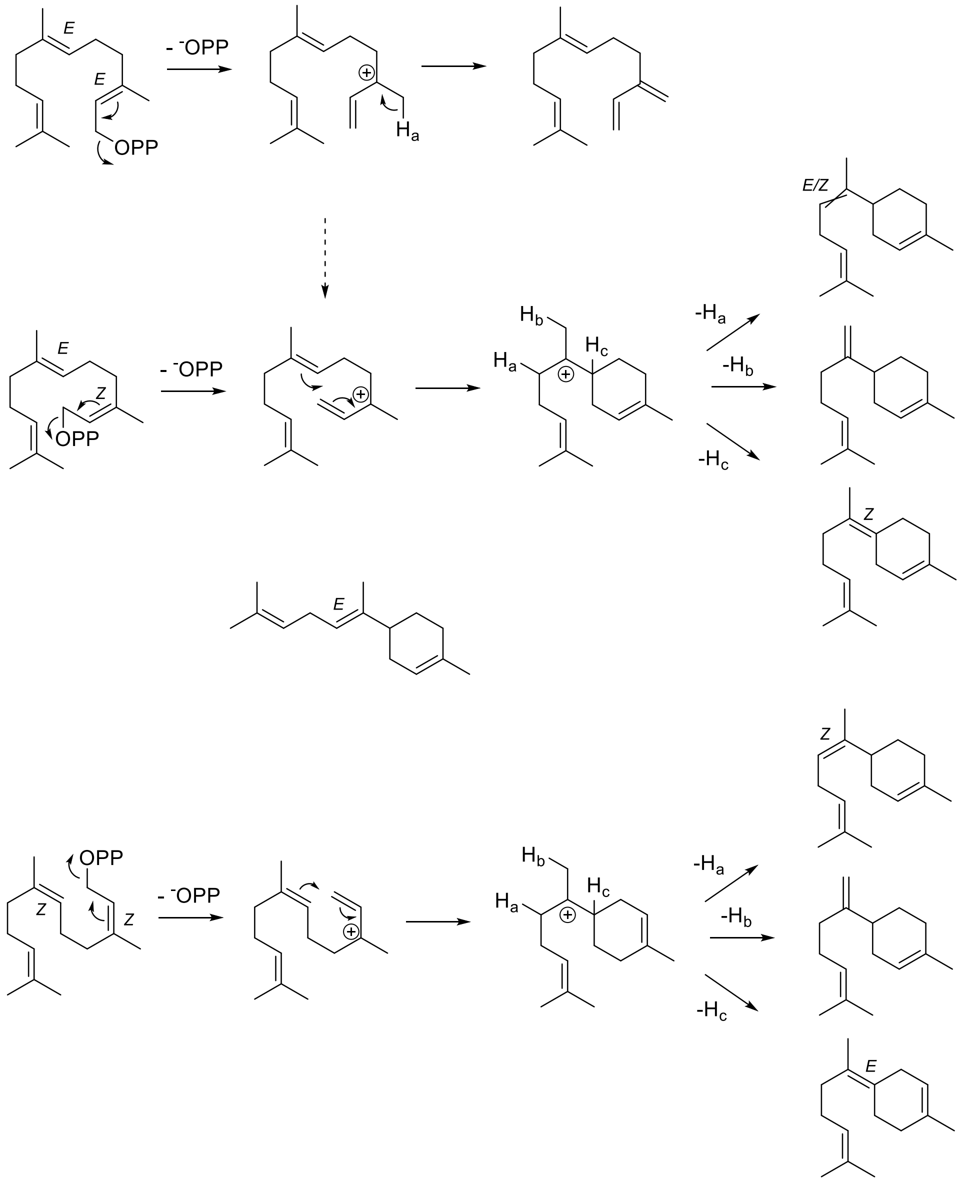

Fig. S13. Possible route to bisabolene isomers from (*E,E*)-, (*Z,E*)-, (*Z,Z*)-FPP.

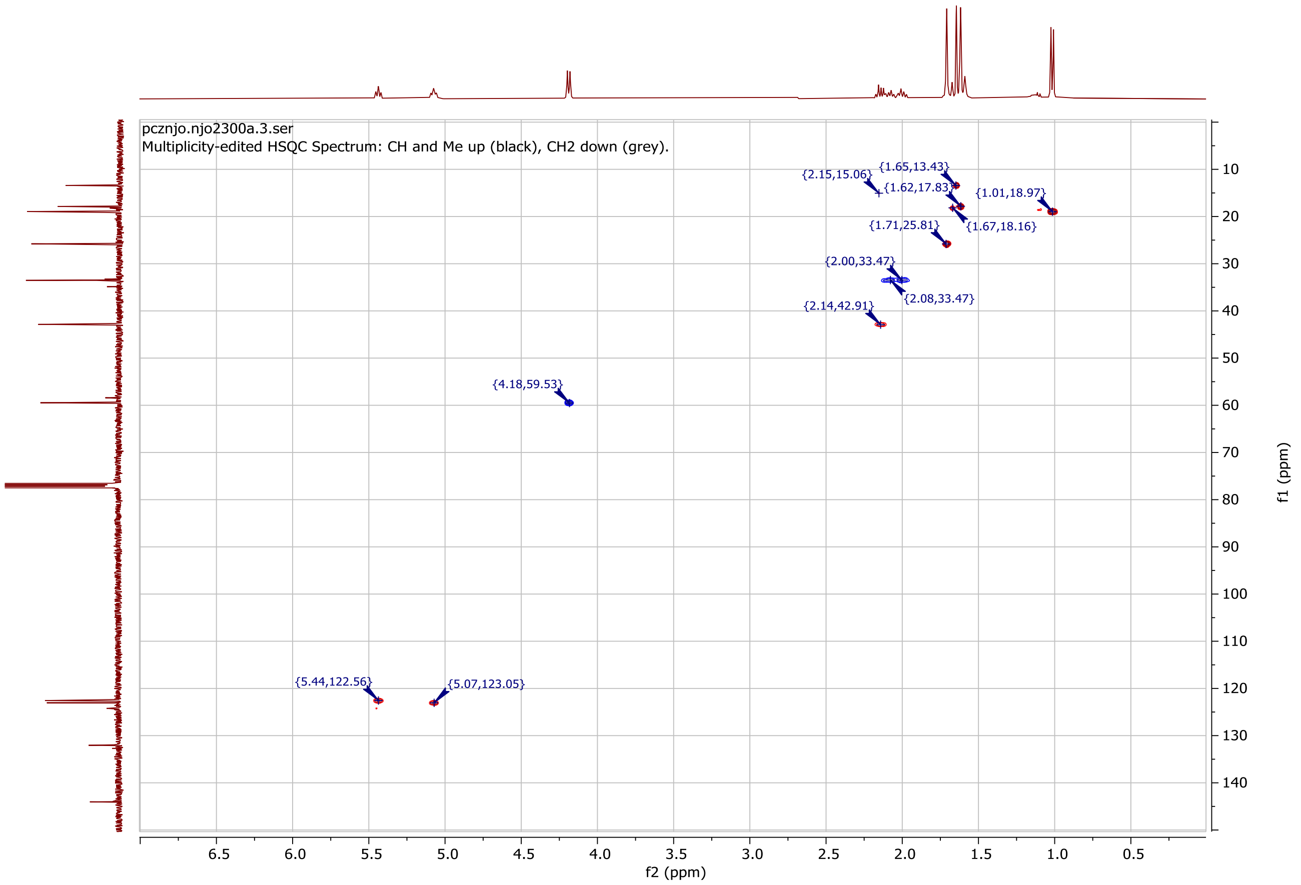

Fig. S14. ^{1}H-^{13}C HSQC NMR spectrum of (±)-4-methylgeraniol.

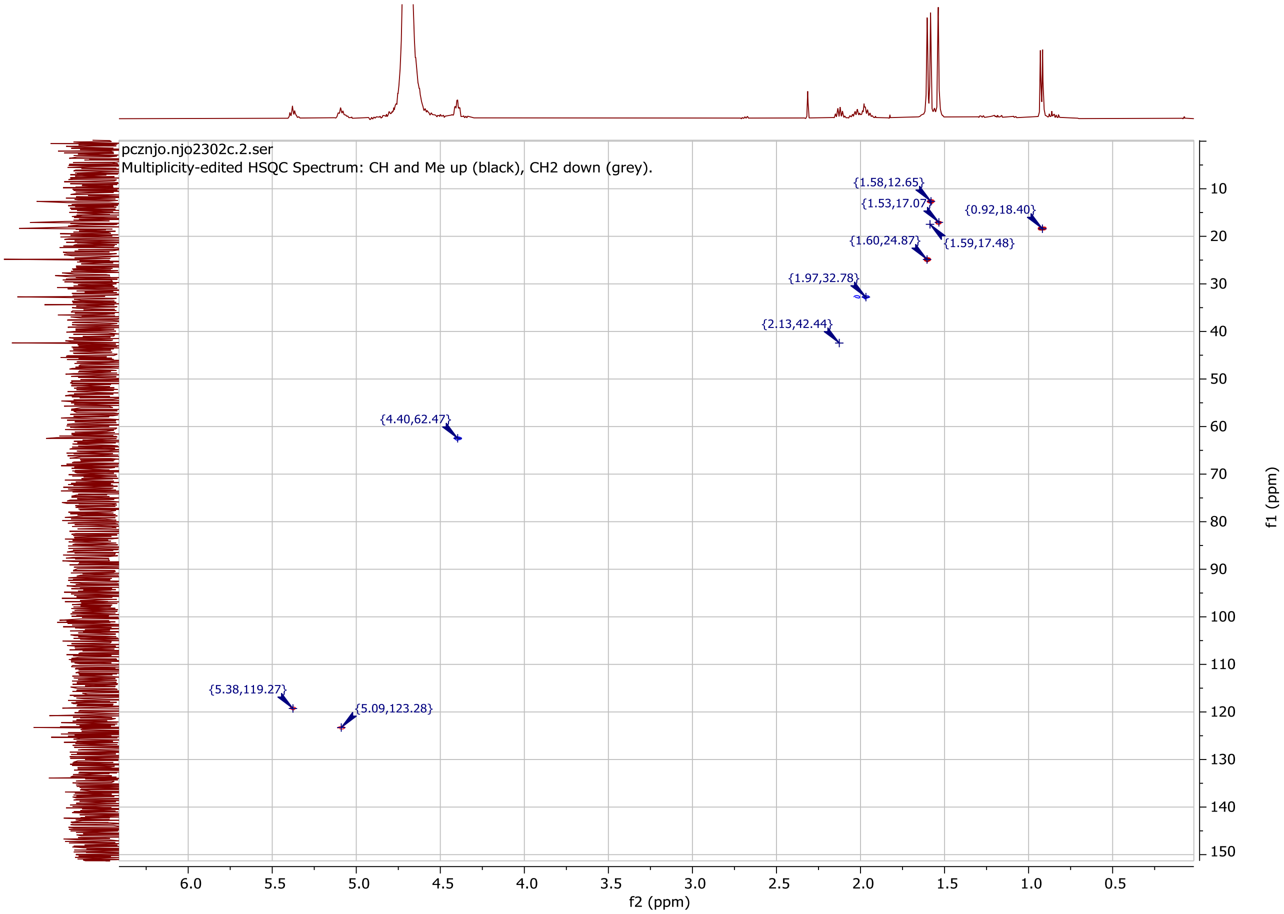

Fig. S15. ^{1}H-^{13}C HSQC NMR spectrum of (±)-4-methylgeranyl diphosphate.

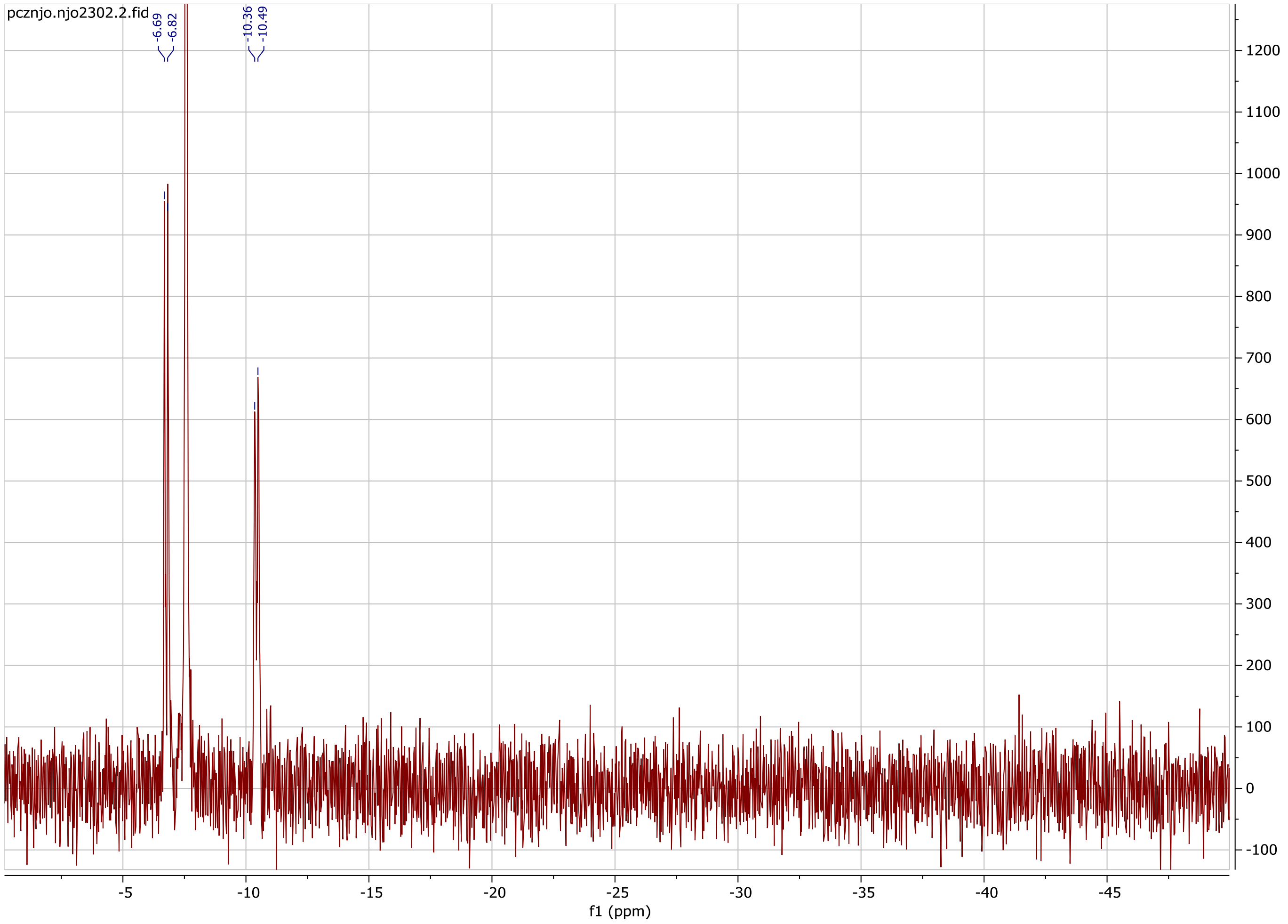

Fig. S16. ^{31}P NMR spectrum of (±)-4-methylgeranyl diphosphate.
